# Accelerated Chemical Reaction Optimization Using Multi-Task Learning

**DOI:** 10.1021/acscentsci.3c00050

**Published:** 2023-04-13

**Authors:** Connor J. Taylor, Kobi C. Felton, Daniel Wigh, Mohammed I. Jeraal, Rachel Grainger, Gianni Chessari, Christopher N. Johnson, Alexei A. Lapkin

**Affiliations:** † Astex Pharmaceuticals, 436 Cambridge Science Park, Milton Road, Cambridge, CB4 0QA, United Kingdom; ‡ Innovation Centre in Digital Molecular Technologies, Yusuf Hamied Department of Chemistry, University of Cambridge, Lensfield Road, Cambridge, CB2 1EW, United Kingdom; § Department of Chemical Engineering and Biotechnology, University of Cambridge, Philippa Fawcett Drive, Cambridge CB3 0AS, United Kingdom; ∥ Cambridge Centre for Advanced Research and Education in Singapore Ltd., 1 Create Way, CREATE Tower #05-05, 138602, Singapore

## Abstract

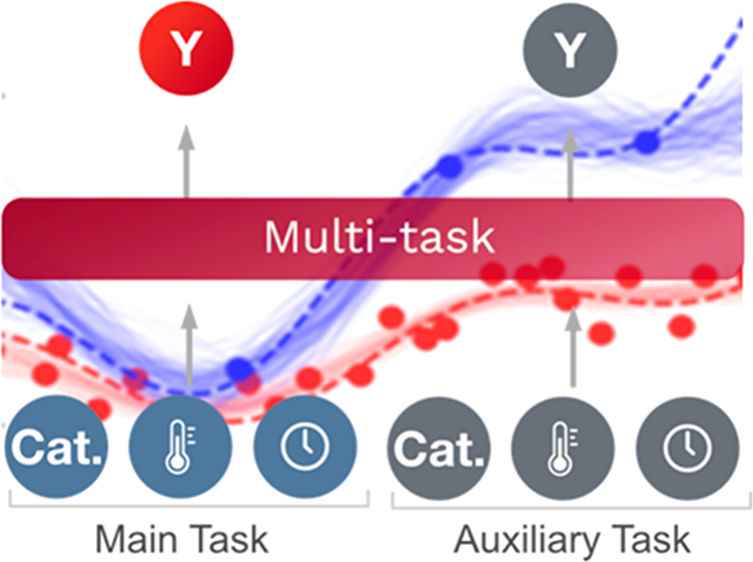

Functionalization of C–H bonds is a key challenge in medicinal chemistry, particularly for fragment-based drug discovery (FBDD) where such transformations require execution in the presence of polar functionality necessary for protein binding. Recent work has shown the effectiveness of Bayesian optimization (BO) for the self-optimization of chemical reactions; however, in all previous cases these algorithmic procedures have started with no prior information about the reaction of interest. In this work, we explore the use of multitask Bayesian optimization (MTBO) in several *in silico* case studies by leveraging reaction data collected from historical optimization campaigns to accelerate the optimization of new reactions. This methodology was then translated to real-world, medicinal chemistry applications in the yield optimization of several pharmaceutical intermediates using an autonomous flow-based reactor platform. The use of the MTBO algorithm was shown to be successful in determining optimal conditions of unseen experimental C–H activation reactions with differing substrates, demonstrating an efficient optimization strategy with large potential cost reductions when compared to industry-standard process optimization techniques. Our findings highlight the effectiveness of the methodology as an enabling tool in medicinal chemistry workflows, representing a step-change in the utilization of data and machine learning with the goal of accelerated reaction optimization.

## Abstract 2

This study represents the use of multitask learning and autonomous experimentation to greatly accelerate reaction optimization for medicinal chemistry applications.

## Introduction

In recent years, there has been an increased interest in the use of automated, self-optimizing continuous flow platforms for the optimization of chemical processes.^1−5^ These platforms use automated reactors and machine-learning algorithms to learn from previous experiments, and thereby choose future experiments that ultimately maximize yield and/or other process objectives. The use of self-optimizing platforms has arisen from the desire for faster reaction optimization, improved process sustainability, and cheaper overall process development.^6−9^ The use of these platforms aims to enhance the capabilities of the researcher by removing the need for repetitive and labor-intensive experimentation, allowing them to focus on more challenging tasks. By leveraging algorithms, the platforms utilize only minimal reaction material but gain the most process information possible, making their deployment in fine chemical and pharmaceutical industries very attractive.^10,11^

Recent work has shown that Bayesian optimization is a particularly powerful tool for self-optimization applications.^12−14^ However, in all previous studies, each optimization begins with no *a priori* information about the chemical landscape for the reaction of interest. This protocol therefore requires initial experimental iterations whereby the algorithm is learning about the experimental design space, without any prior information on where the optimal reaction conditions may be. This initial exploration can be expensive in terms of both cost and time, particularly when there may already be data on the broad chemical transformation of interest from previous optimization campaigns. This also opens the methodology to some uncertainty over which initial design strategy to use, such as forms of factorial design or Latin Hypercube sampling (LHS), which will affect the overall experimental budget. *General* well-performing reaction conditions can also be identified for particular reaction classes, as highlighted in recent work by Angello et al.,^15^ but do not give optimal conditions for specific transformations and cannot account for important parameters such as specific reactor differences, solubility, reaction selectivity, differences in substrate functionality, or further adjacent objectives (E-factor, purification, downstream processing, etc.). The use of optimization strategies for specific substrates in specific instances is therefore still important for determining optimal yields (or other process outputs).

For many medicinal chemistry applications, such as in developing chemistries for the synthesis of potential drug candidates, the use of efficient optimization techniques is paramount due to the minimal quantity and increased preciousness of intermediate reaction materials.^16,17^ This is a particular problem in fragment-based drug discovery (FBDD),^18,19^ as challenging transformations are often required on highly functionalized molecules—including difficult C–C forming reactions utilizing precise C–H activations which ideally must be executable in the presence of polar groups that are required for binding to the target protein.^20^ The excessive material consumption when utilizing existing algorithms may also be a reason that medicinal chemists are less attracted to these cutting-edge optimization techniques than process chemists. Our hypothesis is that optimization strategies that can utilize pre-existing chemical knowledge could mitigate unnecessary material use, accelerate process development, and present the potential for broader applicability in new synthetic chemistry methods.

This work shows the first real-world examples of leveraging previous reaction optimization data for unseen chemical transformations using multitask Bayesian optimization (MTBO), with our prior work on MTBO for chemistry only showcasing its use in a simulated setting.^21^ The framework of MTBO, first introduced by Swerksy et al.,^22^ replaces the standard probabilistic model in Bayesian optimization with a multitask model. As these multitask models can be trained on data from related tasks, we can therefore utilize data from previously conducted similar reactions—both from the laboratory and from the literature. In this work, we first explore and benchmark the use of MTBO in simulated studies, then exploit the methodology to optimize several pharmaceutically relevant C–H activation reactions using an autonomous flow reactor platform. These experimental case studies were chosen to highlight the effectiveness of MTBO in a medicinal chemistry, particularly FBDD, context through efficient material usage. There are many reports of similar automated workflows in the recent literature where a self-optimization protocol is utilized.^23,24^ Our reactor platform is equipped with a liquid handling robot and can optimize both continuous variables (residence time, temperature, etc.) and categorical variables (solvent, ligand, etc.). This ability is seldom reported in the literature (with some notable examples from several research groups^1,25−27^), likely due to engineering and equipment challenges, but is very important in determining all relevant parameters that influence reaction outcomes. The MTBO algorithm utilized is integrated into the open-source reaction optimization package Summit^28^ and represents a powerful data-driven optimization technique that can utilize known reaction data and ultimately lead to savings in material, time, and overall cost.

## Results and Discussion

### Bayesian Optimization to Multi-Task Bayesian Optimization

**Figure 1:**
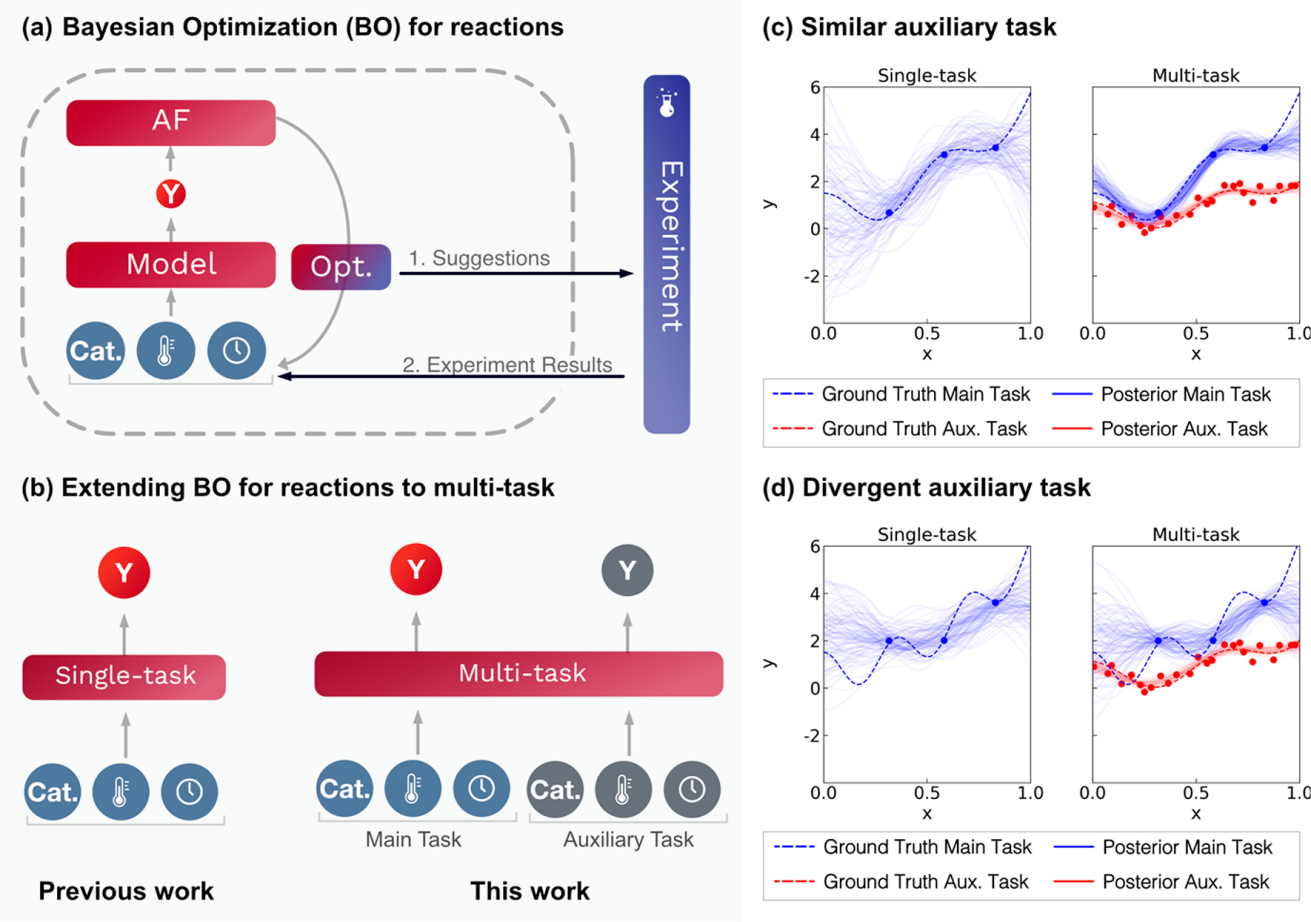
Schematic description of multitask Bayesian optimization to the context of reaction optimization. (a) Bayesian optimization consists of a probabilistic model (typically a Gaussian process) that predicts experiment outputs (e.g., yield) given experiment conditions; an acquisition function (AF) that predicts the value of potential new experiments; and an optimization algorithm (opt). (b) Multitask Bayesian optimization replaces the Gaussian process with a multitask Gaussian process trained simultaneously on an auxiliary task. In our case, this auxiliary task is a similar reaction to the one being optimized, utilizing previous experimental results. (c) When the auxiliary task for a multitask Gaussian process is similar to the main optimization task, predictions on the main task are improved significantly. (d) When the auxiliary task for a multitask Gaussian process is divergent to the main optimization task, predictions on the main task are similar to what is observed for the baseline single-task Gaussian process.

As shown in Figure 1a, Bayesian optimization (BO) relies on three key components: a probabilistic model, an acquisition function, and an optimization algorithm.^29^ The probabilistic model is trained using experimental data and acts as a surrogate or “simulation” of the real chemical reaction. Given this probabilistic model, the acquisition function estimates the values of different potential experimental reaction conditions. The optimization algorithm is then used to find the set of experimental conditions that maximizes the acquisition function, and these experimental conditions are hence suggested as the next real experiment to run. The combination of the probabilistic model and the acquisition function enables exploitation of known high performance areas and exploration of new chemical space. By iteratively executing the suggested experimental conditions, retraining the model and optimizing the acquisition function, the BO protocol progressively identifies the best reaction conditions for the output of interest.

As shown in Figure 1b, MTBO changes the probabilistic model in BO. Typically, a Gaussian process (GP) is used as the probabilistic model in BO due to the general applicability and efficiency of GPs in the small data regime.^30^ MTBO replaces a GP with a multitask GP that can learn the correlations between different tasks to enable better predictions. In our case, the tasks are chemical transformations from the same reaction class with varying substrates. A formal definition of GPs and multitask GPs is in the Methods section.

As a simple illustration of the benefits of multitask GPs, we created example functions with one input and one output, then trained both a GP and multitask GP on only three data points. In the multitask GP case, we also generated 25 data points from an auxiliary task. As shown in Figure 1c, when the main and the auxiliary tasks are similar, the predictions from the multitask GP (shown as samples from the posterior of the GP) more accurately represent the underlying function than the predictions from the single-task GP. The multitask GP leverages covariance between the data in the two tasks to improve predictions on the main task, even with limited data for the main task—this is shown formally in the Methods section. As shown in Figure 1d, when the main and the auxiliary tasks are divergent, predictions from the single-task and multitask GP are highly variable. However, this variability in the multitask GP is still useful because the BO algorithm will explore to better capture the underlying distribution of the main task.

### In Silico Case Studies: Suzuki–Miyaura Couplings

**Scheme 1:**
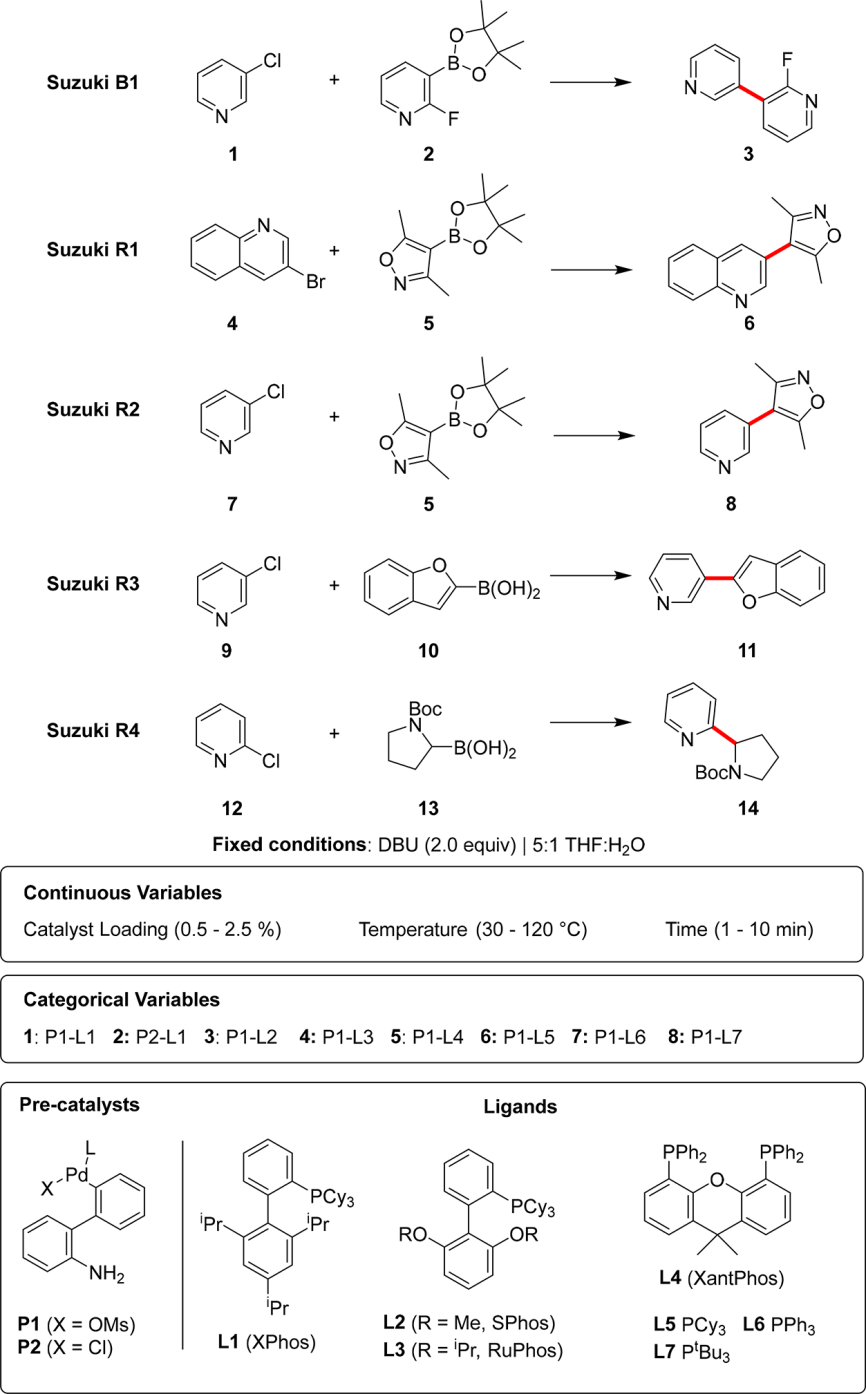
Reactions of Interest for the Suzuki–Miyaura Coupling *In Silico* Case Studies The datasets for training the model Suzuki B1 was taken from Baumgartner *et al*.^31^ and for Suzuki R1-R4 from Reizman *et al*.^32^ In each of these studies, the continuous and categorical variables (with the bounds shown) were optimized for reaction yield.

We first executed *in silico* MTBO studies using model chemical reactions as benchmarks. These models were generated using neural networks trained on literature experimental data that predict reaction yield;^28^ more detail on these models can be found in the Methods section. The model “Suzuki B1” was trained using Suzuki cross-coupling data from Baumgartner et al.,^31^ while the models “Suzuki R1–4” were trained using data from Reizman et al.^32^—these specific transformations and the variables that affect these models are shown in Scheme 1.

**Figure 2:**
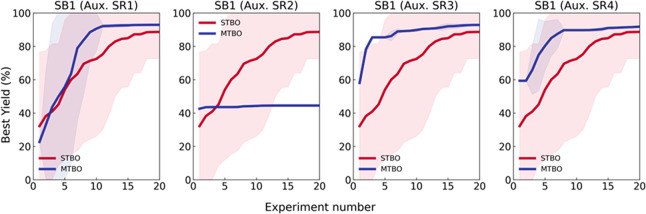
Comparison of the performance of single-task Bayesian optimization (STBO) and multitask Bayesian optimization (MTBO) of Suzuki B1 with Suzuki R1-R4 as auxiliary tasks. The average best yield with a 95% confidence interval over 20 repeats is shown. The label above each plot refers to the auxiliary (Aux.) task based on the names in Scheme 1, where Suzuki is abbreviated to S.

Four specific case studies are highlighted in Figure 2, each where the main task is Suzuki B1 and the auxiliary training task is one data set from each of Suzuki R1–4. In each case study, a conventional single-task Bayesian optimization (STBO) benchmark for the Suzuki B1 reaction serves as a comparison. For each MTBO study, 96 data points from the auxiliary task were utilized. The average best yield for each algorithm is shown with a 95% confidence interval over 20 repeated runs.

When leveraging Suzuki R1 as an auxiliary training task, MTBO initially suggests optimal conditions from the training task with P1-L4 (XantPhos). However, these give very low yields (<25%), which leads to further exploration of the chemical space, resulting in optimal conditions with P1-L1 (XPhos) and a higher yield than STBO. The additional strength shown by MTBO in this case study is the greater speed in obtaining optimal results.

In the second case study, when the auxiliary task is Suzuki R2, MTBO appears to perform poorly—this is likely due to the low reactivity observed in Suzuki R2 and a noisy simulation benchmark (see Figure S8). In this case, the best conditions from the training task also do not perform well on the main task, but the yield is moderate enough that it makes further exploration of the chemical space initially difficult in obtaining a better response. This suggests that MTBO may bias the training data in these circumstances when higher yields are possible but not expected, when given very low-yielding auxiliary tasks.

In the case studies where the auxiliary tasks were Suzuki R3–4, the reactivity of the substrates was much more similar in both the main and the training tasks, leading to similar optimal conditions being found. This means that MTBO achieved better, and much faster, results than STBO in these cases.

**Figure 3:**
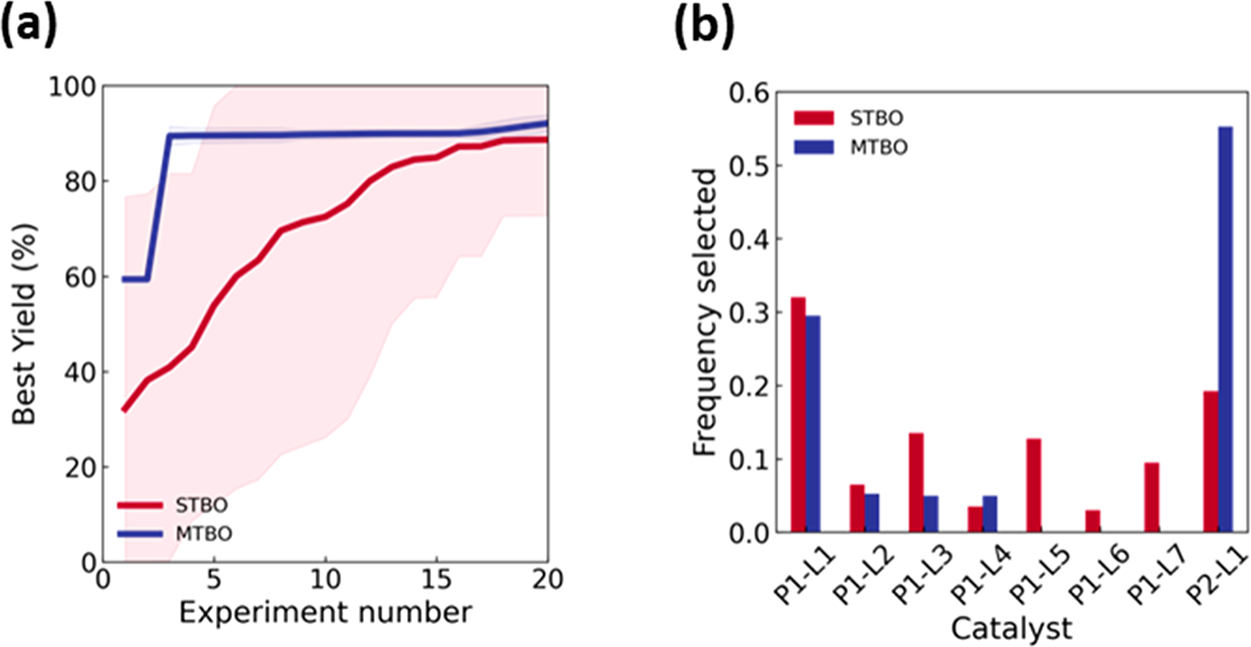
Comparison of the performance of single-task Bayesian Optimization (STBO) and multitask Bayesian Optimization (MTBO) of Suzuki B1 and all of Suzuki R1-R4 as auxiliary tasks. (a) The average best yield with a 95% confidence interval over 20 repeats is shown. (b) Frequency of selection of each catalyst in Scheme 1 by STBO and MTBO.

Performance of MTBO can be greatly improved using multiple auxiliary tasks. As shown in Figure 3a, when Suzuki B1 is optimized with Suzuki R1-R4 as auxiliary tasks, the optimal conditions are always found by MTBO in fewer than five experiments. Both P1-L1 and P2-L1 are considered optimal for this reaction,^31^ and MTBO selects these two catalysts in over 80% of experiments during 20 repeats, when compared to <50% frequency for STBO—this is highlighted in Figure 3b. As MTBO utilizes optimal regions of chemical space that have been identified in previous tasks with similar reactivity, this allows the algorithm to identify new (and better performing) optimal reaction conditions faster.

These simulated case studies suggest that the use of MTBO is often beneficial, particularly when not mapping the predicted reactivity differences of the main and auxiliary substrates *a priori*. Initial guesses (optimization starting points) are typically better than random initialization because of previous reaction information, and the rate of “best yield” improvement is also greater. In the best-case scenario, the reactivity of the new substrate is similar to those of previous data sets and results in a greater yield much faster than standard STBO. In the worst-case scenario, MTBO can fail with one noisy auxiliary case, but we found that using multiple auxiliary tasks helps to mitigate these issues. With these findings, we were confident that MTBO would be effective in real-world case studies where we have experimental data sets from previous optimization campaigns. Further *in silico* case studies for other reaction types, namely, Buchwald–Hartwig cross couplings, were also conducted and showed similarly promising results; these studies can be found in the Supporting Information.

### Experimental Case Studies: C–H Activation

**Scheme 2:**
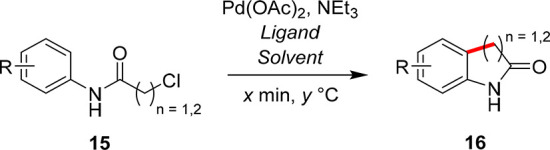
Reaction Class of Interest for the MTBO Study, Where the Substituted Chloroacetanilide, **15**, Reacts to Form the Corresponding Oxindole, **16** Pd(OAc)_2_ and NEt_3_ remain constant in each experiment, but the ligand, solvent, catalyst concentration, residence time, and reaction temperature are optimized for each case study.

The reaction class that we targeted for our experimental MTBO study was the palladium-catalyzed C–H activation reaction, reported by Hennessy and Buchwald,^33^ yielding pharmaceutically relevant oxindoles (**16**) from their corresponding chloroacetanilides (**15**), as shown in Scheme 2. Each case study is shown in Table 1 and is highlighted if it is forming a potential bioactive fragment or active pharmaceutical ingredient (API) intermediate. The rationale behind these studies is 2-fold: first, these oxindoles are closely related to many known bioactive molecules and hence medicinal chemistry projects, and second, when considering optimal growth vectors for bioactive molecular fragments to grow into more potent drug candidates (such as in FBDD),^18^ the most beneficial transformations are often exploiting C–H bonds on the fragment to form new C–C bonds.^20^ Therefore, using MTBO, we aimed to rapidly optimize several transformations using different starting materials with unique functionalities to yield structurally diverse oxindole products by forming valuable sp^2^-sp^3^ C–C bonds. Then, for future optimization campaigns requiring oxindole syntheses, this model can be employed to expediate reaction optimization and process development for new substrates.

**Table 1:**
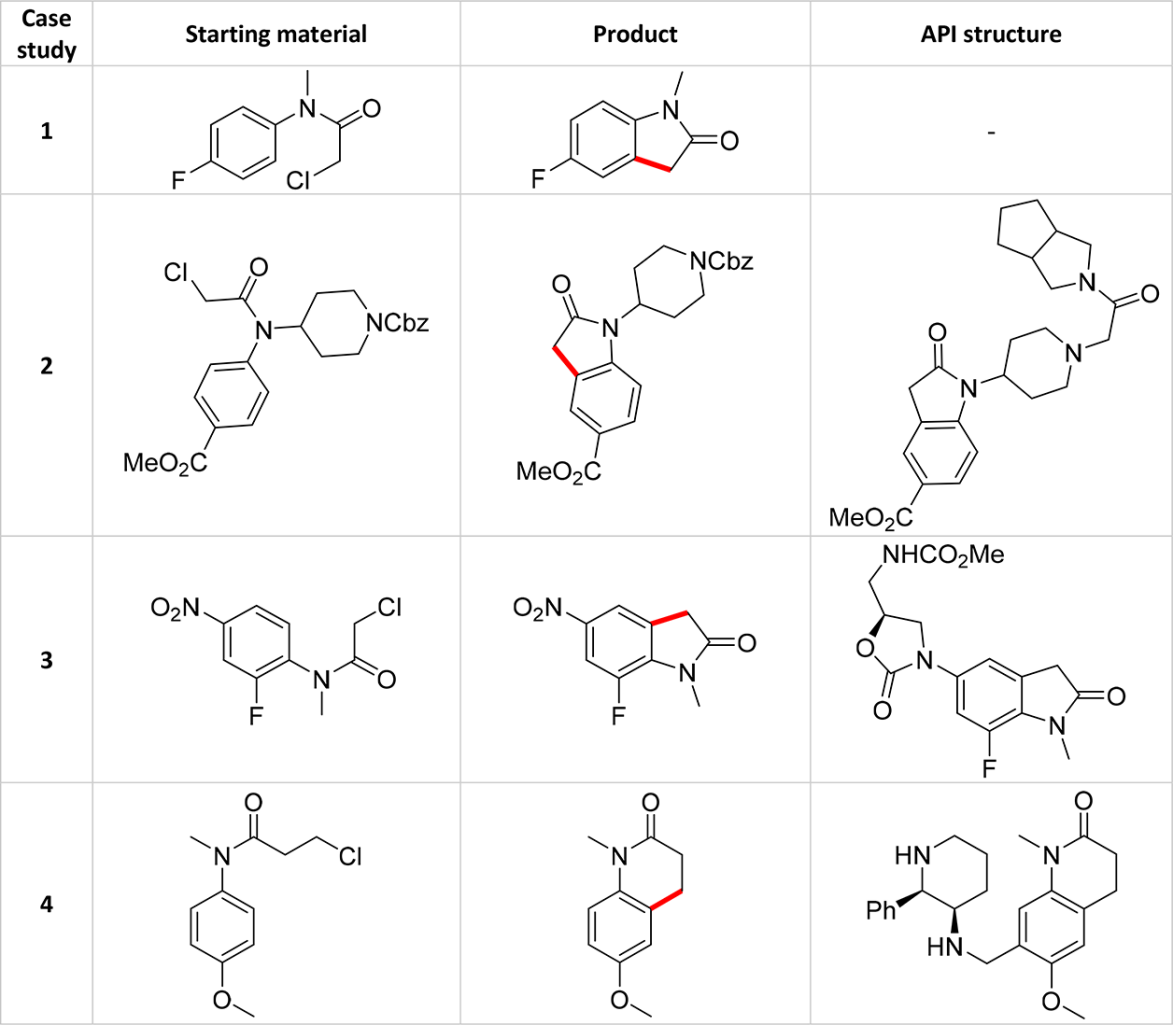
Each Experimental Case Study Explored in This Work, Including the Starting Material Used, The Product Formed and the API Structure That the Product Is Linked to^a^

a These reactions, and references to their known API structure, are highlighted in Schemes 3–6.

**Figure 4:**
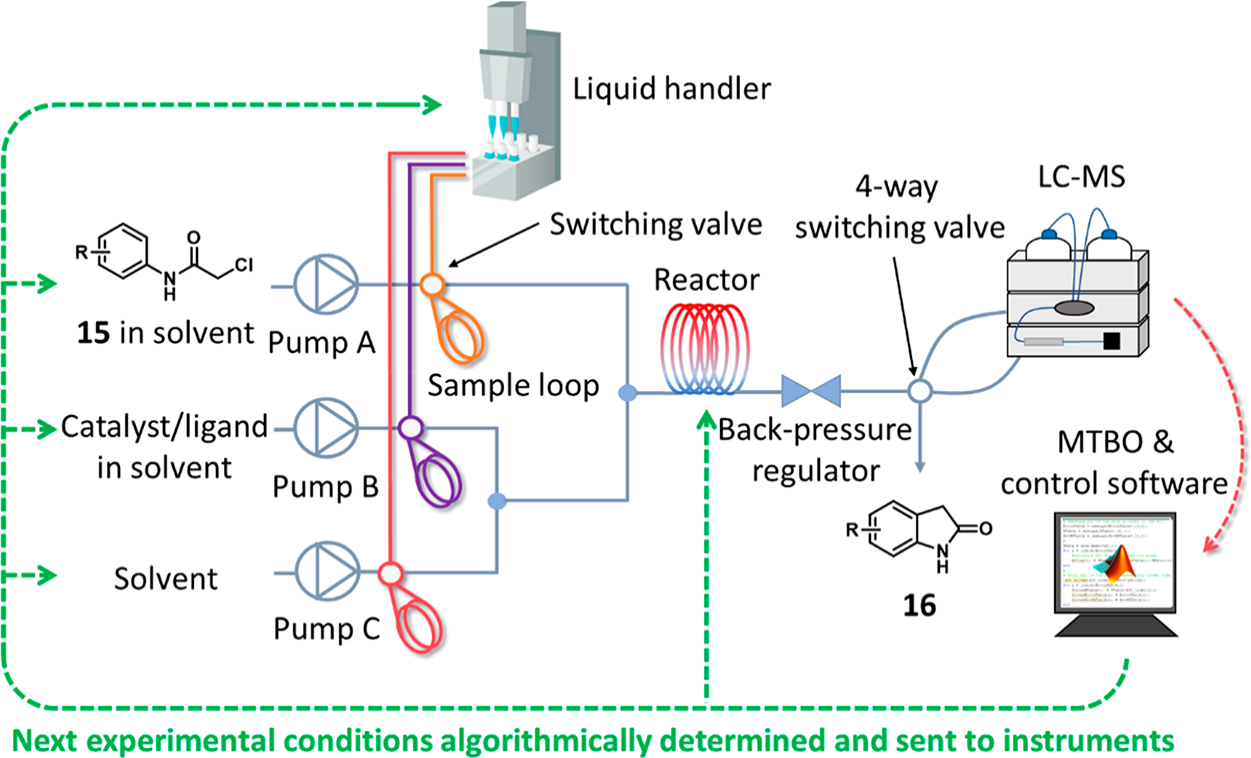
Schematic diagram of the experimental setup and protocol we used for the MTBO self-optimization studies.

For all experimental work conducted during this study, a self-optimizing flow reactor platform was utilized with a control interface previously disclosed by our group.^34^ This platform employs an autonomous optimization workflow, where all experiments are conducted and analyzed without human intervention. All initial training experiments are planned using LHS; then the results from these automated experiments (the yield of the product) are exported using online HPLC sampling. This LHS step is only present when there is no previous experimental information for MTBO to learn from. Based on these reaction data, and any previously conducted auxiliary tasks, the MTBO algorithm then determines the most beneficial reaction conditions to execute in order to maximize product yield. The actual product yield obtained from this reaction is then communicated back to the algorithm, where the experimental feedback loop is closed, as the algorithm suggests conditions for the next optimization iteration (as shown in in Figure 4). Furthermore, only minimal amounts of reaction material are consumed in each experiment by using reaction slugs;^35^ this is an important miniaturization consideration relevant to medicinal chemistry settings, but could potentially be miniaturized further. The minimum slug length is determined on the basis of dispersion in laminar flow such that sampling from a slug is consistent between slugs in repeated tests—the volume of the slugs used in these studies is 4–6 mL. This slug volume is determined by the Vaportec Flow Commander software and varies depending on the necessary solvent dilution. The aim of this experimental methodology is to accelerate the optimization timeline by requiring fewer experiments and less reaction material consumption. More information on the reactor setup can be found in the Methods section.

**Scheme 3:**
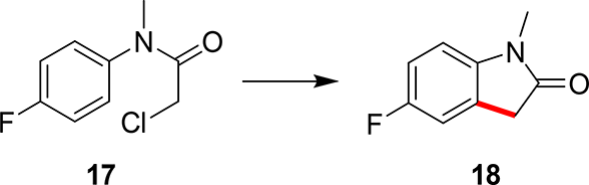
First Case Study Explored Using STBO, Where the Substituted Chloroacetanilide, **17**, Reacts to Form the Oxindole, **18** This product is previously unreported via this C–H activation methodology.

For each case study, we optimized the continuous parameters: residence time (5–60 min), reaction temperature (50–150 °C), and catalyst concentration (1–10 mol %), and the categorical parameters, solvent (toluene, DMA, acetonitrile, DMSO, NMP) and ligand (JohnPhos, SPhos, XPhos, DPEPhos), for the maximum product yield output. While it is possible to represent these categorical variables in numerous ways, the simplest representation (one-hot encoding) proved sufficient to learn from.^36,37^ The first case study, as shown in Scheme 3, utilizes only single-task Bayesian optimization (STBO) as there is no previous data to leverage model understanding for MTBO. The starting material, **17**, reacts to form the molecular fragment (with potential growth vectors for further functionalization), **18**. The optimization was initialized using 16 (2^4^) training experiments before the algorithm began to suggest experimental conditions.

**Figure 5:**
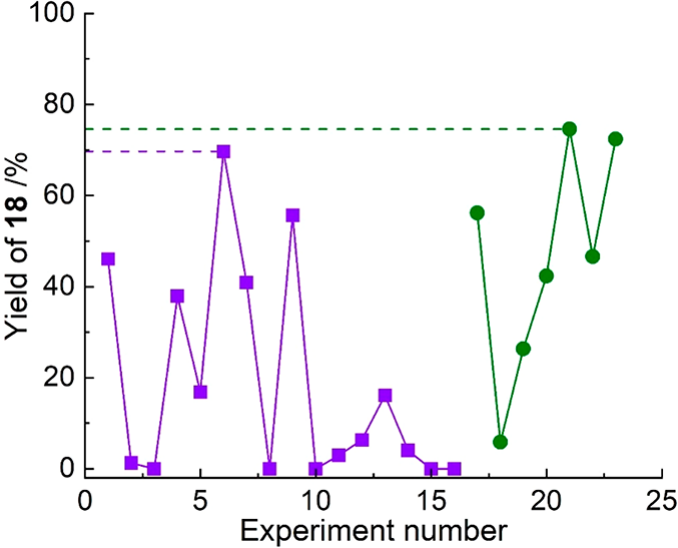
Plot of yield of product, **18**, against experimental number in the STBO campaign, where purple ■ = training experiments and green ● = optimization experiments.

After the initial training experiments, the feedback loop (as described in the Methods section) was implemented and 7 further experiments were conducted, finding the optimal reaction parameters of: NMP, XPhos, 53 min residence time, 89 °C reactor temperature with 9 mol % catalyst, yielding the product, **18**, in 74.6% yield. These results are interesting, because with many reported optimization campaigns the optimal conditions are often the most forcing (highest temperature, highest residence time, highest catalyst concentration).^3,5,38^ However, in this case, the algorithm determines that a moderate reactor temperature is important for a higher yield. This is because the starting material reacts to form other products, leading to a decrease in the desired product yield under more forcing conditions. Furthermore, the optimized conditions reported in the original publication describing these types of reactions feature toluene and JohnPhos,^33^ which are different from our optimized parameters for this reaction. However, these reported conditions require reaction times of 2.5–6 h which are difficult to replicate in flow, which could be the reason the same categorical parameters were not determined to be optimal in our 5–60 min residence time optimization space. A plot of the experimental data, both training and optimization experiments, and the yields achieved are shown in Figure 5. These 23 experiments required to achieve optimal conditions are also significantly fewer in number than what would be required for current industrial-standard optimization procedures, such as design of experiments (DoE), which would require >750 experiments of efficient design space exploring data points. All reaction data for each case study is reported in full in the Supporting Information, as well as efficiency comparisons with industrial-standard optimization procedures.

**Scheme 4:**
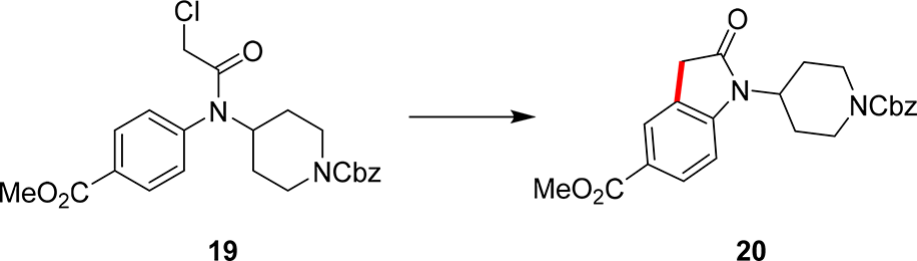
Second Case Study Explored Using MTBO, Where the Substituted Chloroacetanilide, **19**, Reacts to Form the Key Intermediate En Route to a Serine Palmitoyl Transferase (SPT) Inhibitor, **20** This product is previously unreported via this C–H activation methodology.

Utilizing the experimental data set from this optimization campaign, a different substrate was then explored in the second case study. The starting material, **19**, reacts to form a key intermediate for a serine palmitoyl transferase (SPT) inhibitor, **20**, as shown in Scheme 4.^39^ As this is a similar transformation, the use of MTBO should hasten optimization and produce optimal reaction conditions much more quickly. The optimization is initiated, and the first suggested experiment deviates only slightly from the previously obtained best parameters, while still utilizing NMP and XPhos as the categorical variables but produces a poor yield of the product (14.8%). As this yield is much lower than what the underlying multitask model had predicted, the corresponding weightings to select this area of parameter space for this case study are greatly reduced and thereby the likelihood of exploring this area again during this campaign is reduced. The model then balances the exploration of new parameter space with the exploitation of known favorable conditions, particularly from the previous case study, to iterate through further experiments. The optimal reaction conditions were found in 11 experiments: acetonitrile, JohnPhos, 28 min residence time, 127 °C reactor temperature with 5 mol % catalyst, yielding the product, **20**, in 84.9% yield. It is important to note that this area of parameter space is far from the identified optimum in the previous case study, showing the adaptability of MTBO to similar optimization tasks without simply exploiting near the previously obtained optimal conditions. To identify these process parameters, this entire workflow consumed only 980 mg of the starting material, **19**, and has a much greater throughput (requiring less catalyst loading, cheaper materials, and noncomplex solvent mixtures) than other reports of this chemistry that yield only 76% of the desired product.^40^ This experimental data is displayed at the end of this section in Figure 5 (red dotted line).

With two completed optimization campaigns, these data sets could then be leveraged for the optimization of process parameters for a third case study. This case study features the transformation of **21** into the antibacterial intermediate, **22**, necessary for the synthesis of the oxazolidinone antibiotic Linezolid,^41^ as shown in Scheme 4.

**Scheme 5:**
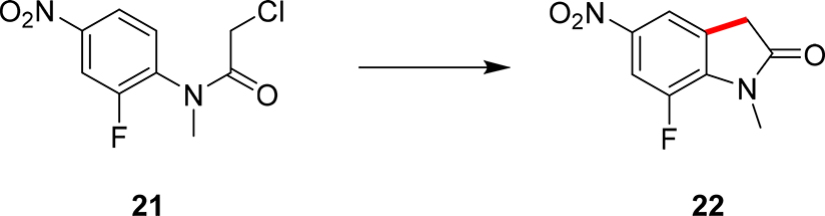
Third Case Study Explored Using MTBO, Where the Substituted Chloroacetanilide, **21**, Reacts to Form the Key Intermediate, **22**, for the Antibiotic Linezolid This case study utilized data from the previous two optimization campaigns. This product is previously unreported via this C–H activation methodology.

The initial experiment in MTBO used similar conditions to the optimal conditions from the second case study, with acetonitrile and JohnPhos as categorical variables with 18 min residence time with 5 mol % catalyst at 139 °C. This produced a good yield of 71% but was subsequently improved by using NMP and XPhos, as the MTBO algorithm discovered from the first case study is also a parameter space region of high interest, immediately improving the yield to 83%. Upon further adjustment of the continuous variables, a yield of 98% was achieved in only five total experiments. This is the first optimization campaign where one ortho site was blocked for cyclization, but this variation is seemingly not enough to divert chemical reactivity from what the MTBO algorithm expects, thereby proving the task’s applicability to these divergent structures. The entire workflow for optimizing this process used only 250 mg of the starting material, **21**, which also resulted in a greater yield, throughput, and greener process than other reports in the literature (86% yield in batch, overnight using fluorinated solvents).^42^ This experimental data is displayed at the end of this section in Figure 5 (orange solid line).

**Scheme 6:**
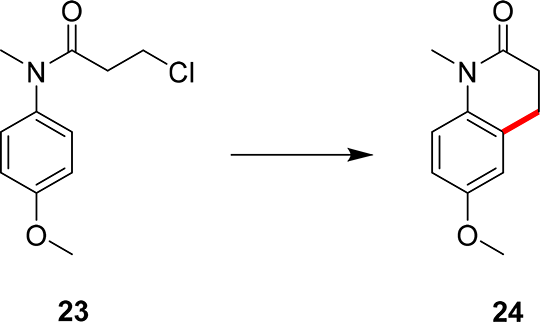
Another Case Study Explored Using MTBO, Where the Substituted Chloroacetanilide, **23**, Reacts to Form the Pharmaceutical Intermediate, **24**, for the Synthesis of an NK1 Receptor Antagonist. This case study utilized data from each of the previous optimization campaigns. This product is Previously Unreported via This C–H Activation Methodology

The next experimental case study features the transformation of the starting material, **23**, into the NK1 receptor antagonist intermediate, **24**, as shown in Scheme 6.^43^ In this optimization campaign, the MTBO algorithm leveraged data from the previous three case studies; yet this is the first substrate that forms a 6-membered cyclized ring instead of the typical 5-membered ring in the previous oxindoles. Initial experiments in previously identified well-performing parameter space produced low yields, but the algorithm could thereby determine that further exploration of the parameter space was important as the substrate showed more variability from the previous tasks.

Through further iterations, the categorical variables were exploited: DMSO and DPEPhos, with the most forcing continuous parameters: 60 min residence time with 10 mol % catalyst loading at 150 °C. These were determined to be the optimal conditions as found by the self-optimization workflow, giving the product in 82% yield in 10 experiments using only 450 mg of the starting material, **23**. Despite this functional change, the algorithm was still able to determine the optimal conditions utilizing previous data and quickly found that although a similar reaction task was present, further exploration of the parameter space was necessary. This further shows the adaptability of the MTBO approach to wider substrate scopes with different functionalities. This experimental data is displayed at the end of this section in Figure 5 (green dashed line).

A final case study was then attempted using this workflow, which is the same oxindole-forming C–H activation reaction conducted in every other reaction, but this time featured an electron-rich aromatic ring rather than an electron-deficient ring. The substrate of interest, *N*-methyl-2-methylchloroacetanilide, also had one ortho position blocked for cyclization. This study was conducted to further test the limits and the adaptability of the MTBO algorithm, but even with the most forcing conditions possible using our workflow we could only achieve a 29% product yield. This was also true when using the reported categorical conditions for this substrate in the initial publication^33^—however, the differences between the reactor systems may have negatively affected the yield outcome, i.e., 6 hour reaction times cannot be achieved easily in flow. Given these observations, we concluded that the reactivity of this species is sufficiently different to previous case studies and therefore cannot be considered as a similar task to the other optimization campaigns. Therefore, for the optimization of these substrates (or any substrates sufficiently different to the tasks of interest) further MTBO campaigns must be conducted for the models to encapsulate these differences to efficiently optimize any case study of interest. With the addition of computational characterization of each substrate (for example, using DFT or reaction similarity scoring), all substrates of interest can be categorized *a priori* into their respective task bins, avoiding the necessity for additional experimentation. It may also be appropriate in such cases that promising upper bounds leading to full conversion of starting materials are identified, potentially avoiding wasteful experiments in inaccurately defined parameter spaces. Further experimental information on this case study can be found in the Supporting Information.

**Figure 6:**
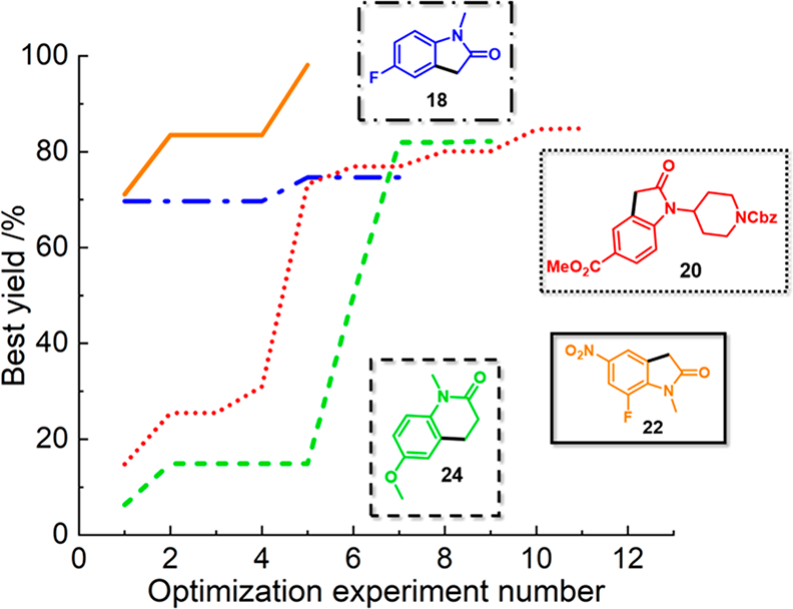
Plot of best yield of the products in each case study against the optimization experimental number in each campaign. The initial training experiments for case study 1 are not plotted. The color and dash type of the graph correspond to each product molecule: case study 1 (blue dash-dot), case study 2 (red dot), case study 3 (orange solid), and case study 4 (green dash).

For each of these consecutive C–H activation case studies, iteratively fewer experiments were (generally) necessary to achieve an optimal set of reaction conditions for the highest process yields—this is illustrated in Figure 6. This is because there was an increasing data density that detailed optimal areas of parameter space for similar tasks (reactions of similar substrates), allowing for a progressively more efficient optimization workflow. In each case study, only minimal amounts (for our specific reaction system) of starting materials were consumed to find optimal reaction conditions, which is very important in early stage medicinal chemistry development applications when preservation of precious starting materials and speed of optimization are paramount. Other common optimization strategies, such as traditional one-factor at a time (OFAT) approaches, may provide modest process improvements in these scenarios but have been shown repeatedly to underperform when compared with statistically based techniques.^1,44,45^ This methodology has therefore proven to be effective in real-world pharmaceutical applications for material and cost efficiency, with the bonus of full automation that allows scientists to use their human resources to focus on other areas of chemical development. Although these experimental studies focused on C–C bond formation by targeting C–H activation, these techniques can be utilized for other transformations to ultimately accelerate optimization.

## Conclusions

The studies performed in this work, both *in silico* and in real-world chemical applications, represent the first use of data sets from similar reactions to expediate current optimization campaigns with multitask Bayesian optimization. This methodology drastically shortens optimization timelines for pharmaceutically relevant transformations, whereas other traditional process optimization techniques (i.e., design of experiments, kinetics studies) would require a significantly higher investment in starting materials, time, and cost—principally because of the large, nonlinear design space introduced alongside a high number of categorical variables. This would likely make their optimization infeasible in medicinal fragment-to-lead/FBDD workflows and early stage process development, unless using intuition-based optimization techniques (such as OFAT) that are unlikely to obtain optimal results.^1^ By introducing more miniaturization technology, including smaller reactors/slugs and plate-based screening, there is the added opportunity to reduce material consumption even further using these automated platforms.

With the increasing density of chemical reaction data, both in the literature and in private data storage, there is a wealth of information that can be leveraged for building task-specific models to further increase the efficiency of future reaction optimizations. When using these multitask learning approaches, it is possible to generate sets of models for specific reaction classes (e.g., Buchwald–Hartwig, Suzuki, etc.) and subsets of those models (electron-rich, sterically hindered. etc.) to rapidly optimize any transformation likely to be encountered. This is a particularly powerful technique in cases where starting materials are sparse and the reaction is poorly understood, yet suitable quantities of product are required for further molecular design, functionalization, and biological testing. Similarly, this importance is echoed in early process development when scale-up of a novel synthetic intermediate is required from the milligram scale to multigram or kilogram scale. The primary challenge when using multitask Bayesian optimization is its tendency to bias toward the best conditions found in a single auxiliary task, as shown in our *in silico* studies. However, our results demonstrate that additional useful auxiliary tasks can reduce the impact of a noisy, low-yielding auxiliary task. Future work could use a more exploratory acquisition function in combination with the multitask model to strike the right balance between biasing toward the auxiliary task data and exploring untested conditions.

The multitask Bayesian optimization algorithm used in this study is open-source and is released as a package within the Summit framework previously reported by our group.^28^ This step toward utilizing machine learning and previous reaction data for future optimization campaigns will ultimately result in faster and more efficient optimizations, thereby serving as a broadly applicable enabling tool with relevance to medicinal chemistry and FBDD settings, where industry-standard process optimization techniques are impractical or even impossible to implement.

## Methods

### Flow Reactor Platform

The reactor platform consists of two Vaportec R2 modules for controlling flow rates, a Vaportec R4 reactor module for controlling reactor temperature, a Gilson GX-271 liquid handler for dispensing and collecting reaction material, and LC-MS analytical equipment (Shimadzu/Waters) for reaction outcome determination. The Vaportec R2 modules are connected using 30 cm sections of 1 mm ID stainless steel tubing and T-pieces, entering a Vaportec stainless steel reactor (10 mL volume), and exiting via a 50-bar back pressure regulator and a 80 cm section of 1 mm ID stainless steel tubing to a switching valve. For each reaction, with the experimental conditions determined through LHS or algorithmically, the liquid handler dispenses 2 mL slugs of the starting material (in this case, the chloroacetanilide **15**) predissolved in the selected solvent into the sample loop for pump A—this solution also contains biphenyl as an internal standard. The selected catalyst/ligand combination in the same solvent is then loaded into the sample loop for pump B, and the solvent of interest is loaded into pump C for dilution. The reaction is conducted with a constant 0.09 M reactor concentration, yielding the corresponding product (in this case, the oxindole **16**), which is thereby analyzed utilizing a 4-way switching valve^46^ for online LC-MS. Using this methodology, experiments can be run using only minimal amounts of reaction material for each experiment as we are utilizing reaction slugs. This experimental workflow is illustrated in Figure 4.

### Gaussian Processes

For single-task Bayesian optimization, we leverage a Gaussian process (GP) as the probabilistic model in BO due to its excellent performance in the limit of small of data.^30^ A GP is a stochastic process characterized by a mean μ_θ_(*x*) and covariance function *k*_θ_ (*x*,*x*′). The covariance function is often called a kernel, which is the term we will use henceforth.
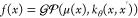
where θ are referred to as hyperparameters of the kernel. Given a finite set of *N* inputs  that correspond with outputs  the GP is a multivariate Gaussian distribution:
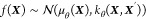


The mean function and kernel act as a prior on the GP. μ_θ_(*x*) is usually set to zero because the kernel *k*_θ_ (*x*,*x*′) fully expresses any arbitrary function. In this work, we use the Matérn 5/2 kernel, with hyperparameters θ = {σ, ***L***}.  is the scaling hyperparameter, and  is a length scale that indicates the significance of each input feature.
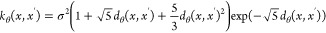
where *d* is the Euclidean distance weighted by the length scale:
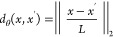


Inference on the GP is done by calculating the posterior of the GP. The posterior of the GP is also a Gaussian distribution:
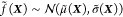

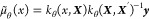

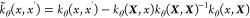
where σ̃(*x*) are the diagonals of the covariance matrix calculated using *k̃*_*θ*_(*x*,*x*′). To train the GP, the log likelihood is maximized, which is the probability that the model predicts the training outputs given the inputs and hyperparameters. The log likelihood avoids overfitting by trading off accuracy of fit to the training data and complexity of the model. The optimal hyperparameters θ* are found by maximizing the log likelihood of the outputs *y* given the inputs ***X*** and the hyperparameters θ^47^ (where Σ_θ_ = *k̃*_*θ*_ (***X***,***X***′)):
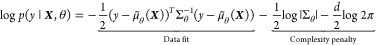


### Multitask Gaussian Processes

Multitask GPs can be used on multioutput functions *f*:χ → *R*^*T*^, where each of the *T* outputs can be seen as solutions to unique regression tasks. The key idea is to use a kernel that can extend to multiple tasks. As detailed in the work by Bonilla et al.,^48^ we use the intrinsic model of coregionalization, which transforms a latent function to yield the outputs:
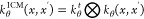


The task kernel *k*_θ_^*t*^ is a *T* × *T* matrix of trainable parameters where *T* is the number of tasks. These parameters represent the intertask correlation.

### Bayesian Optimization

Bayesian optimization aims to solve the optimization problem:
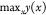
where *y*(*x*) is the underlying function that we observe via experiments. We use the expected improvement (EI) acquisition function for *in silico* experiments^49^ or q-noisy expected improvement (qNEI) acquisition function for flow chemistry experiments.^50^

In BO with EI as an acquisition function, the aim is to choose the point that is expected to improve the most upon the existing best observed point y* ≥ *y* (*x*_*i*_)∀*i* ∈ (1, ···, *t*) where *t* is the number of observations thus far. Therefore, we create an improvement function *I*(*x*) describing the improvement of the posterior of the GP over the best observed point. If there is no improvement, *I*(*x*) = 0.
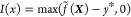


After several manipulations, a closed form of EI can be found:
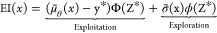
where .

EI suffers from issues with noisy experiments due to its reliance on the best observed point y*, which is a biased estimate, especially in the low data regime. qNEI aims to overcome this issue by using the maximum of posterior of the GP over the observed inputs:^50^
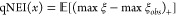
where *ξ*_obs_ ∼ *f̃*(*x*) and *ξ*_obs_ ∼ *f̃*(***X***) are samples from the posterior of the GP. We use BOtorch for implementations of GPs and Bayesian optimization.^50^ For the experimental C–H activation case studies shown in Schemes 4–6, the qNEI acquisition function was used, while EI was used in the simulation case studies due to computational limitations.

### Benchmarks

Prior to real experimentation, we wanted to understand the performance of MTBO in simulated studies. We examined two literature reports that contain experimental results from Suzuki–Miyaura coupling reactions^31,32^ and one report with results from a Buchwald–Hartwig cross-coupling^51^ (demonstrated in the Supporting Information), building a predictive model for the reaction yield to behave as the ground-truth for simulated optimization studies. Buchwald–Hartwig and Suzuki–Miyaura couplings are ubiquitous in the pharmaceutical and fine chemicals industries as they allow rapid construction of aromatic scaffolds through reactions with few impurities.^52^ We therefore chose these reaction classes because of their high value and applicability to real-world scenarios. More details on benchmark training can be found in the Supporting Information.

## Supporting Information Available

Benchmarking of algorithmic procedures using literature data, all experimental data from the optimization campaigns, and a safety statement (PDF)
Video 1: Experimental setup (MP4)

## Accession Codes

The code for this project can be found at https://github.com/sustainable-processes/multitask.
