# Supplementary material for: Accelerated Chemical Reaction Optimization Using Multi-Task Learning
Source: ACS Cent Sci. 2023 Apr 13;9(5):957–68. doi: 10.1021/acscentsci.3c00050 (PMC10214532; doi:10.1021/acscentsci.3c00050)
Supplement: Supplementary file 1 — oc3c00050_si_001.pdf [file oc3c00050_si_001.pdf]

Accelerated Chemical Reaction Optimization using Multi-Task Bayesian Optimization

Connor J. Taylor[1,2‡*], Kobi C. Felton[‡3], Daniel Wigh[2,3], Mohammed I. Jeraal[4], Rachel Grainger[1], Gianni Chessari[1], Christopher N. Johnson[1], Alexei A. Lapkin[2,3,4*]

[1]Astex Pharmaceuticals, 436 Cambridge Science Park, Milton Road, Cambridge, CB4 0QA, UK. [2]Innovation Centre in Digital Molecular Technologies, Yusuf Hamied Department of Chemistry, University of Cambridge, Lensfield Road, Cambridge, CB2 1EW, UK. [3]Department of Chemical Engineering and Biotechnology, University of Cambridge, Philippa Fawcett Dr, Cambridge CB3 0AS, UK. [4]Cambridge Centre for Advanced Research and Education in Singapore Ltd., 1 Create Way, CREATE Tower #05-05, 138602, Singapore.

‡Authors contributed equally.

*Corresponding author email: C.Taylor@compunetics.net, aal35@cam.ac.uk.

Contents

1. Benchmarking using Literature Data 1
   1.1 Data extraction workflow 2
   1.2 Benchmark Training 4
   1.3 Benchmarking Simulation Details 8
   1.4 Suzuki Benchmarks 9
   1.5 C-N Benchmarks 11
   1.6 A note on literature data for optimization 12
2. C-H Activation Case Studies 12
   2.1 General procedure for synthesis of product analytical standards 12
   2.2 Experimental setup 13
   2.3 Case study 1 15
   2.4 Case study 2 17
   2.5 Case study 3 19
   2.6 Case study 4 20
   2.7 Case study 5 20
3. Safety statement 22
4. References 22

1. Benchmarking using Literature Data

We used literature data to benchmark our Bayesian optimization strategies *in silico*. The challenge is that chemical data is often recorded in unstructured text documents such as journal publications and patents. While there are some conventions, each author is free to express the details of a chemical reaction as they wish. Such unstructured information is not amenable to most machine learning algorithms. Therefore, we designed a custom data extraction workflow that we think is a model for how to apply transfer learning in chemistry.

1.1 Data extraction workflow

As shown in Figure S1, we developed a benchmarking workflow that converts unstructured data into benchmarks that can used for comparing various strategies. We leveraged the Open Reaction Database (ORD) format as a common representation of reactions.[1] We wrote converters from spreadsheet formats to ORD. Once the data was transformed into ORD, the data was loaded into local storage on disk for featurization. Subsequently, the featurization step turned the ORD schema into a set of features that can be used for training a benchmark or a GP for Bayesian Optimization. We used one-hot encodings to represent the categorical variables.

We utilize data from publications on Suzuki couplings (see main text Scheme 1) and C-N couplings (see Scheme S1).[2, 3]

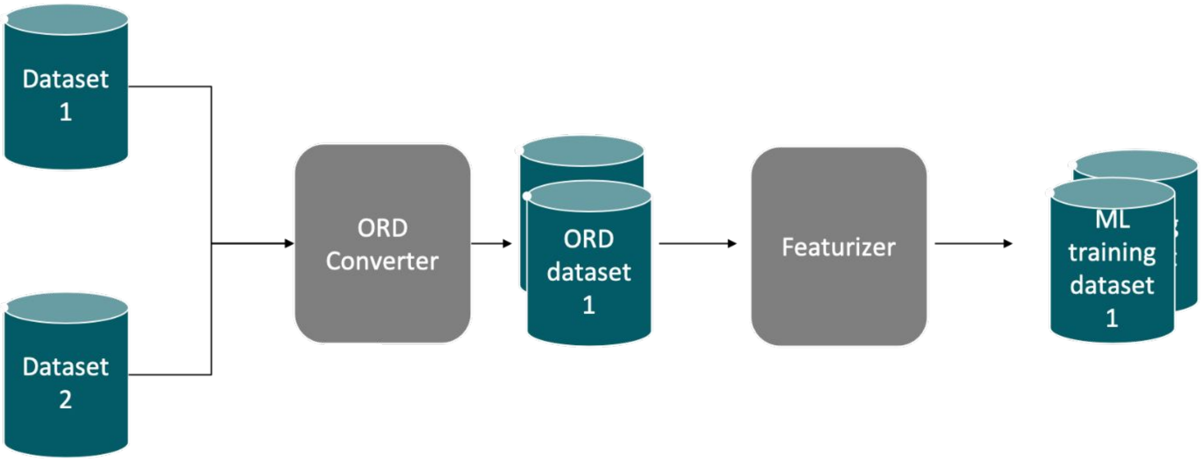

Figure S1: Workflow for converting data in spreadsheets into ORD format and subsequently training datasets for machine leaning.

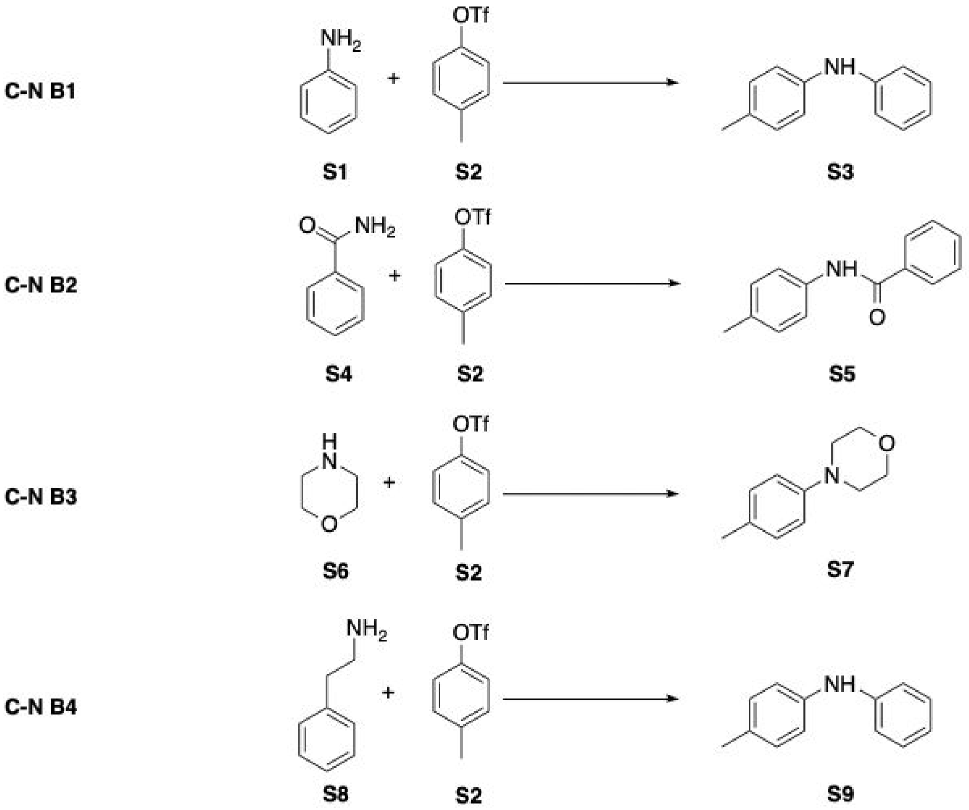

Optimization Variables

Bases: TEA, TMG, BTMG, DBU

Catalysts: P1 - P3

Continuous Variables: Base equiv. (1.0 - 2.5), Temperature (30 - 100 °C), Time (1 - 25 min)

Fixed conditions: 2-MeTHF (or DMSO for C-N B2) I 1.1 mol % catalyst

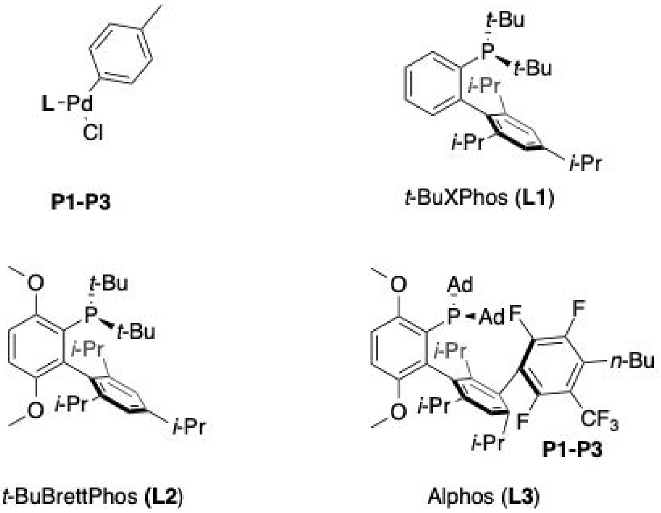

Scheme S1: Benchmark examples of C-N cross coupling (C-N B1-B4) of nitrogen functionalized aromatics (**S1, S4, S6, S8**) with *p*-tolyl triflate. Data is based on data Baumgartner *et al.*[2] The base equivalents, temperature and reaction time, base and catalyst are varied to maximize yield.

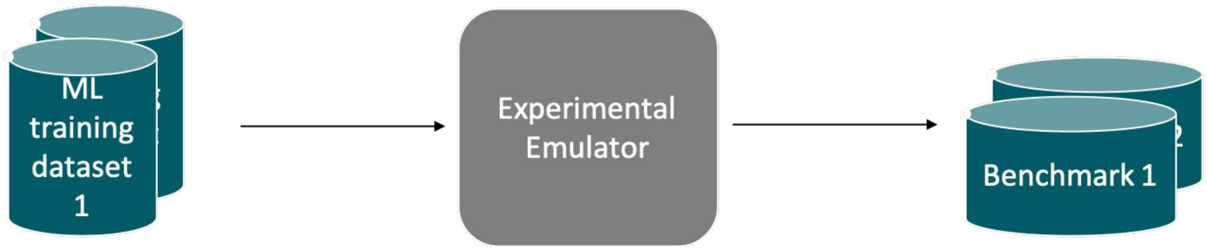

Figure S2: Workflow for training an *ExperimentalEmulator* to act as a benchmark.

1.2 Benchmark Training

We leveraged Summit[4] to build the predictive models from the literature reports. As shown in Figure S2, we utilized the *ExperimentalEmulator* feature in Summit, which creates a benchmark based on experimental data. The regressor used was a neural network with one hidden layer of 512 units with a ReLu activation function. A one-hot encoding was used for the pre-catalyst and ligand combinations. The neural networks were trained by five-fold cross validation over 1000 epochs using stochastic gradient descent. Figures S3 - S10 show the parity plots for the benchmarks.

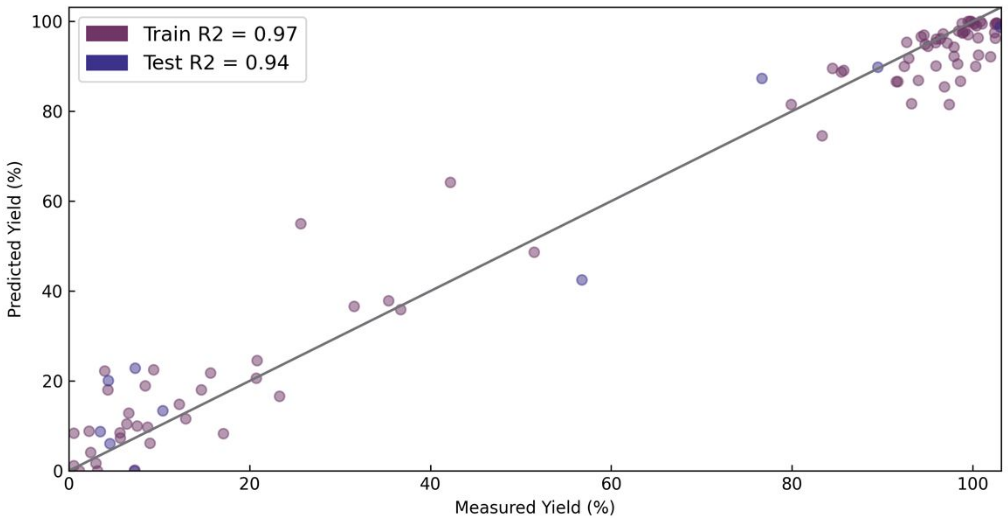

Figure S3: Parity plot of benchmark training for prediction of reaction yield for C-N B1.

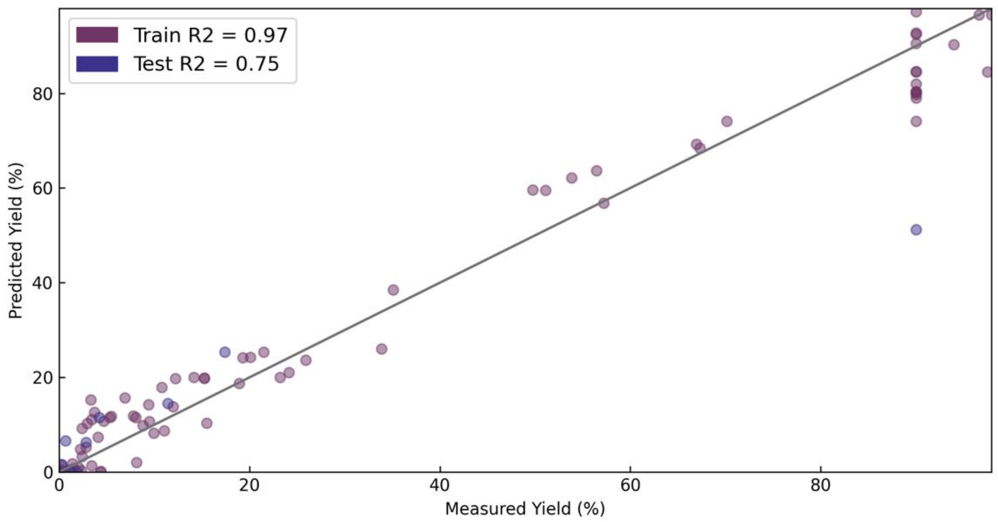

Figure S4: Parity plot of benchmark training for prediction of reaction yield for C-N B2.

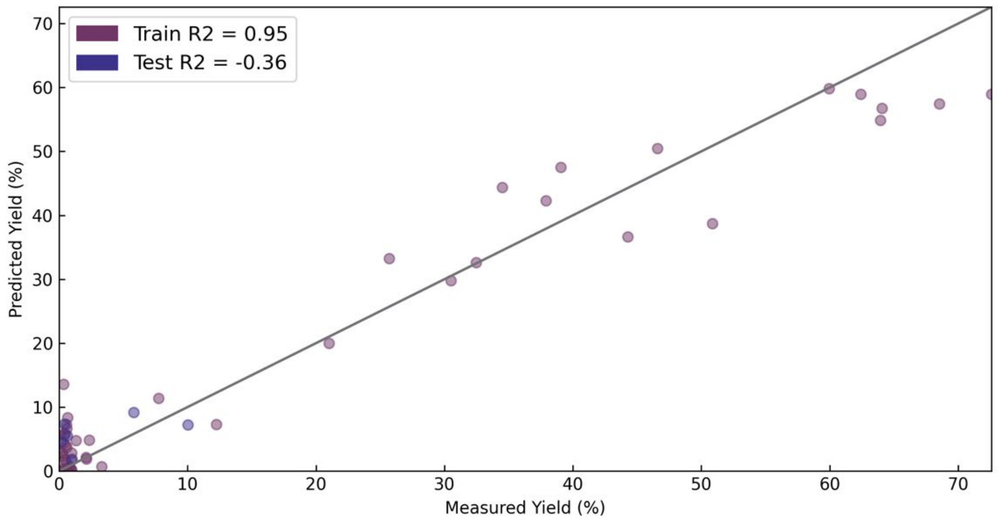

Figure S4: Parity plot of benchmark training for prediction of reaction yield for C-N B3.

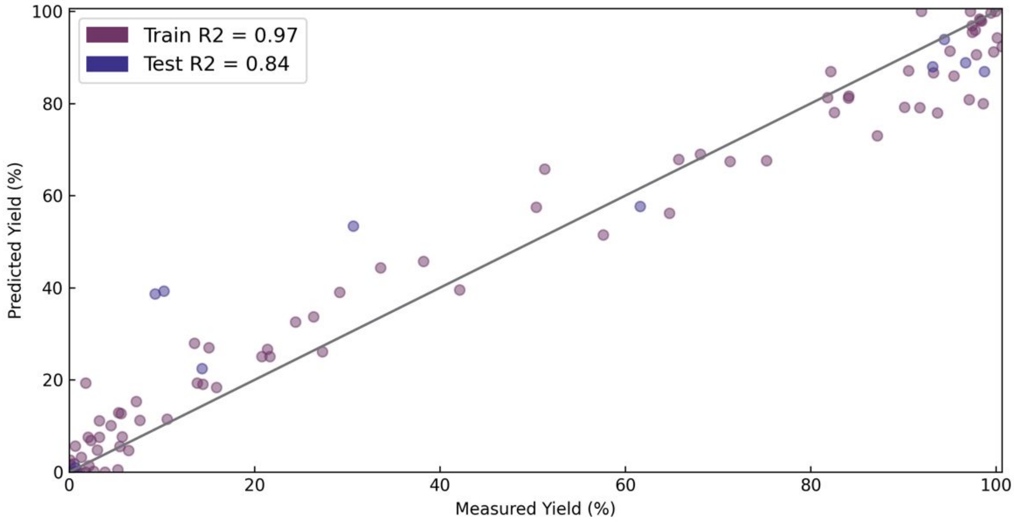

Figure S5: Parity plot of benchmark training for prediction of reaction yield for C-N B4.

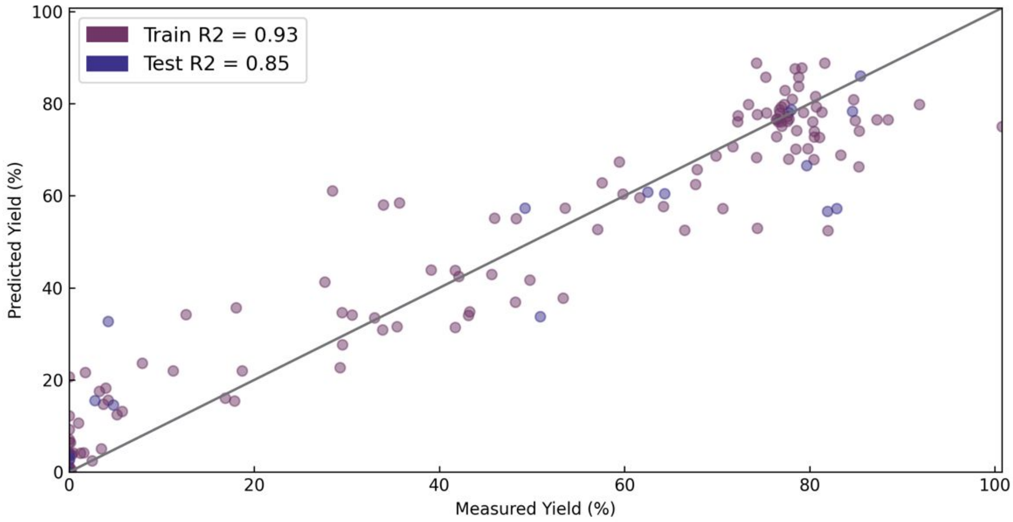

Figure S6: Parity plot of benchmark training for prediction of reaction yield for Suzuki B1.

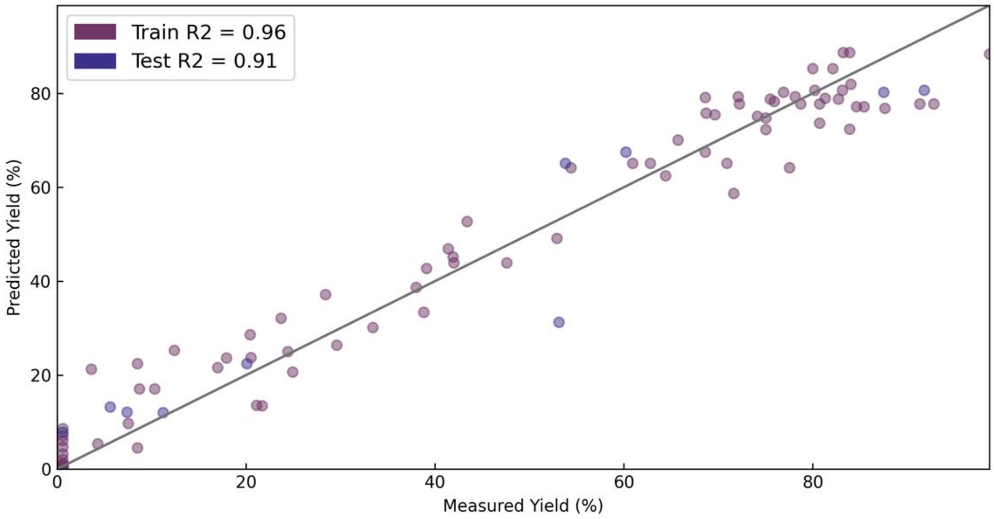

Figure S7: Parity plot of benchmark training for prediction of reaction yield for Suzuki R1.

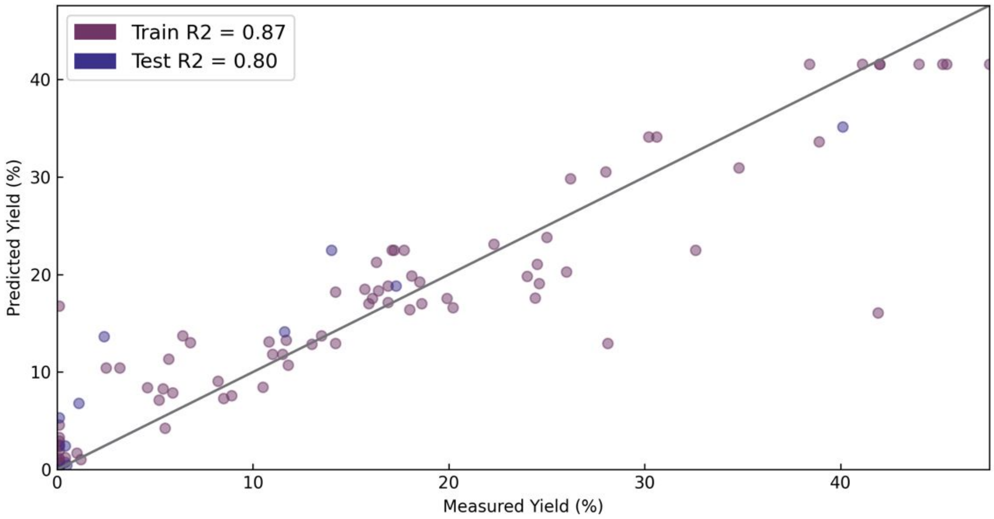

Figure S8: Parity plot of benchmark training for prediction of reaction yield for Suzuki R2.

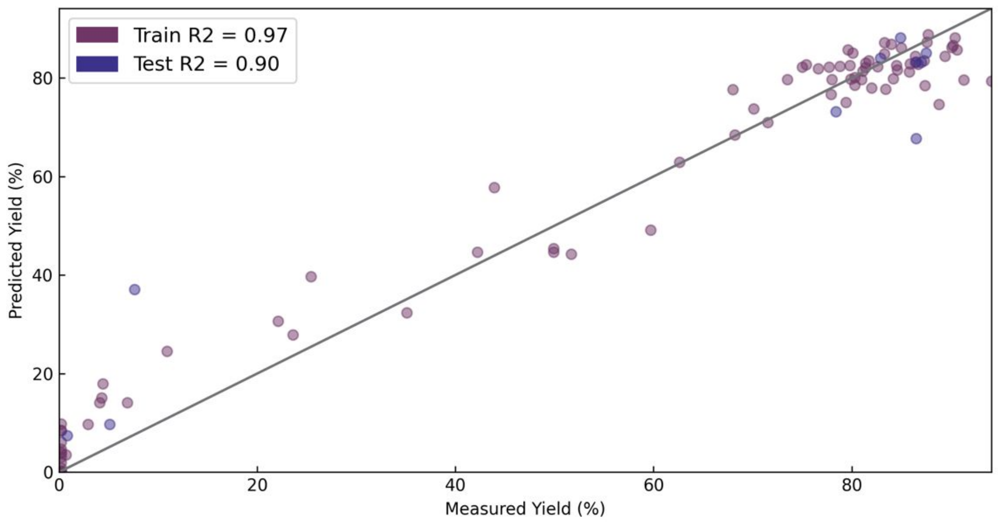

Figure S9: Parity plot of benchmark training for prediction of reaction yield for Suzuki R3.

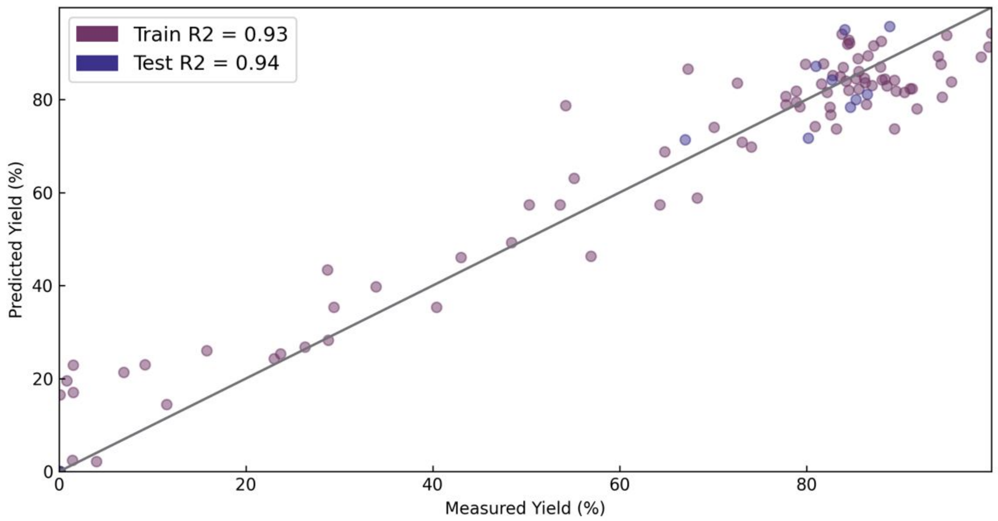

Figure S10: Parity plot of benchmark training for prediction of reaction yield for Suzuki R4.

1.3 Benchmarking Simulation Details

All benchmark simulations were executed on an Amazon Web Services instance with an Nvidia T4 GPU via Lightning AI. For each configuration of optimization and task and auxiliary task, 20 repeats were completed. Figures show the average performance and the 95% confidence interval at each interval. For multitask benchmarks, the first experiment executed was the highest yielding condition from the auxiliary task(s).

1.4 Suzuki Benchmarks

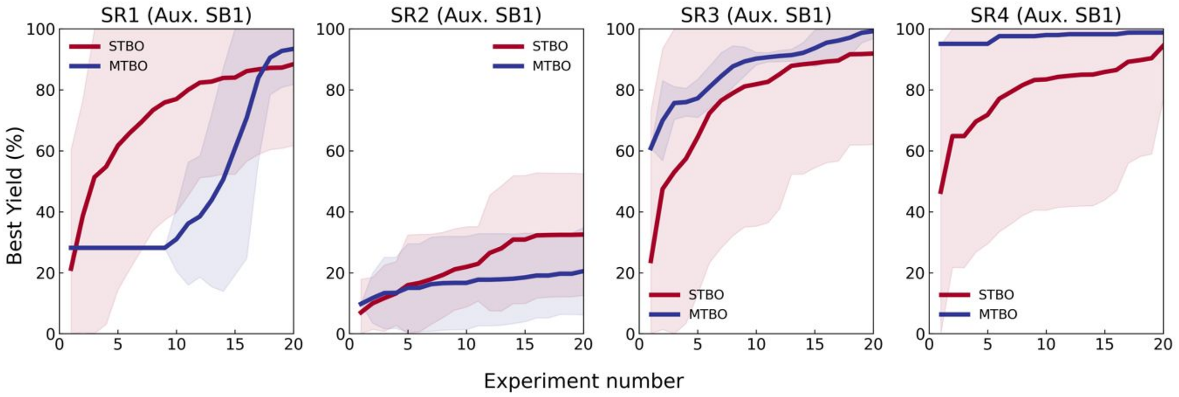

Figure S11: Comparison of the performance of single-task Bayesian optimization (STBO) and multi-task Bayesian optimization (MTBO) on Suzuki reactions R1-R4 with auxiliary data from Suzuki B1.

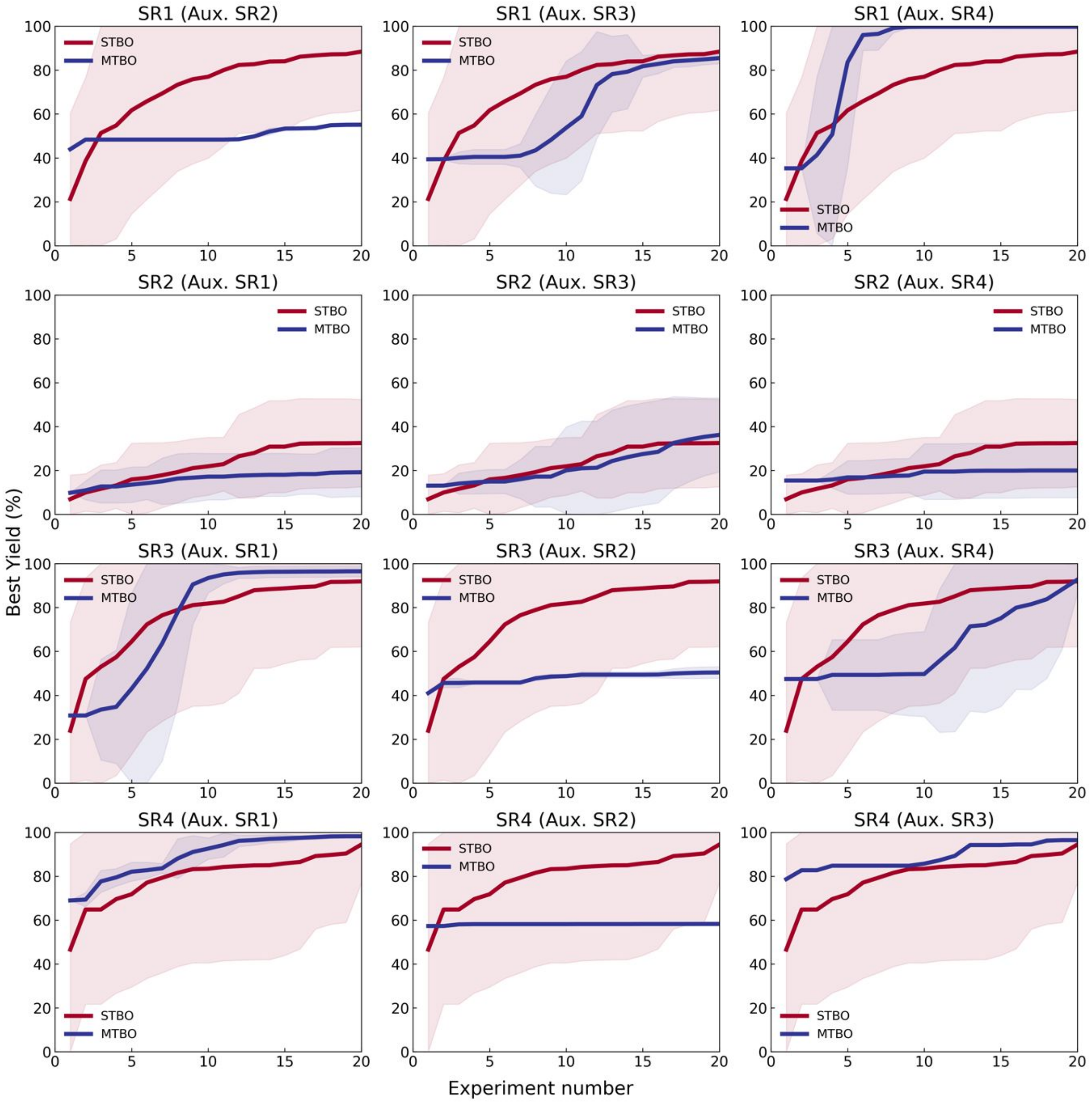

Figure S12: Comparison of the performance of single-task Bayesian optimization (STBO) and multi-task Bayesian optimization (MTBO) on Suzuki R1-R4 with Suzuki R1-R4 as auxiliary tasks. The text above the plot represents the data used as an auxiliary task.

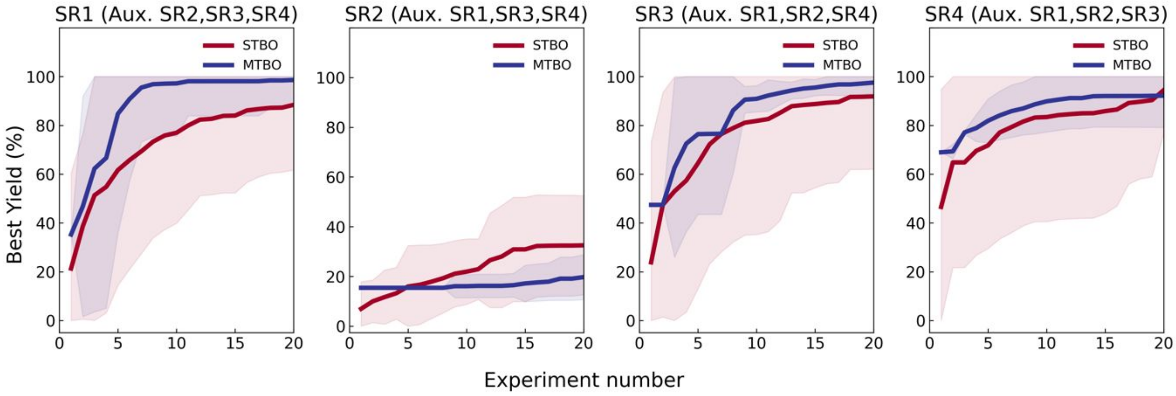

Figure S13: Comparison of the performance of single-task Bayesian optimization (STBO) and multi-task Bayesian optimization (MTBO) on Suzuki R1-R4 with Suzuki R1-R4 as auxiliary tasks. The text above the plot represents the data used as an auxiliary task.

1.5 C-N Benchmarks

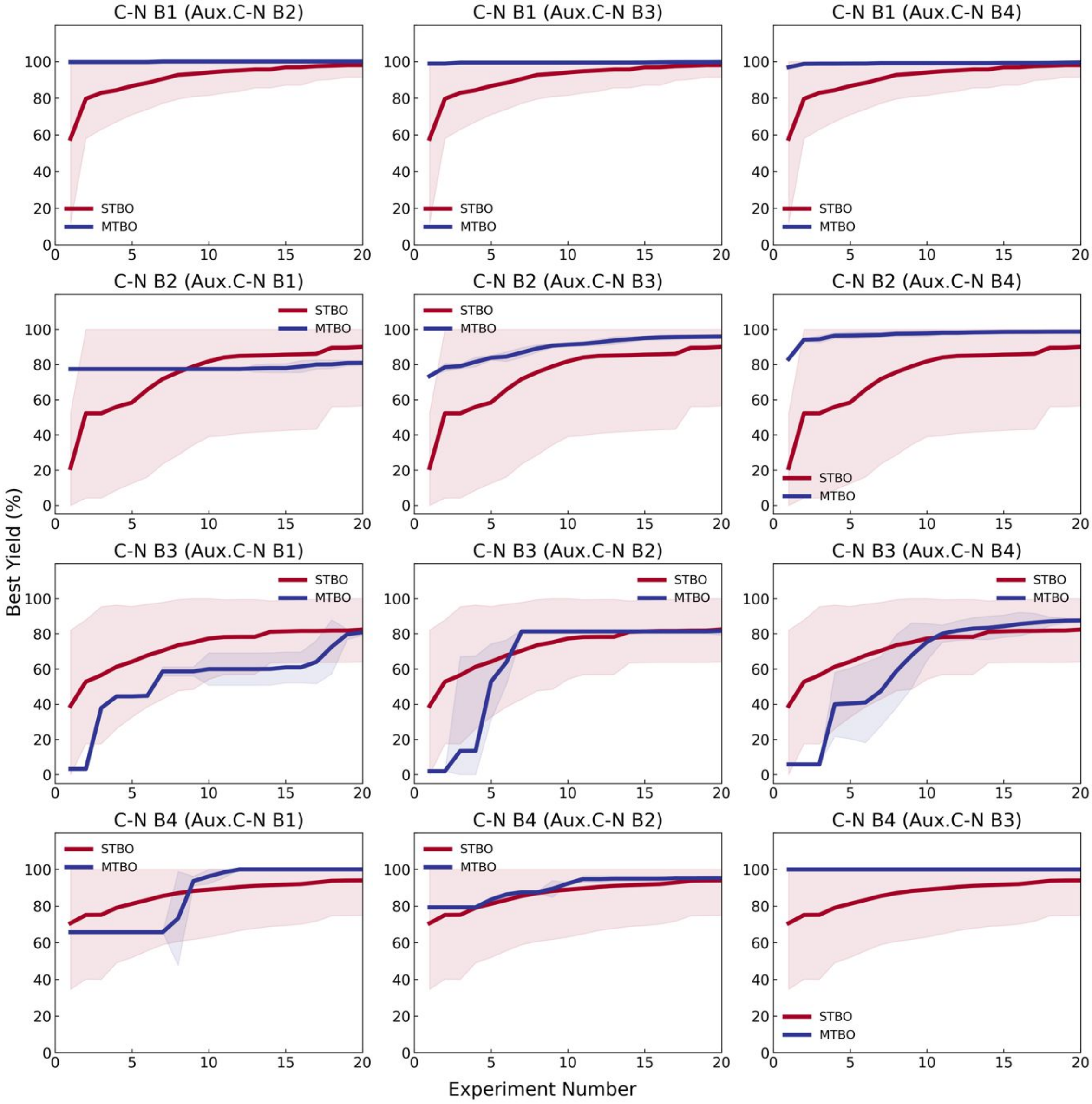

Figure S14: Comparison of the performance of single-task Bayesian optimization (STBO) and multi-task Bayesian optimization (MTBO) on C-N B1-B4 with C-N B1-B4 as auxiliary tasks. The text above the plot represents the data used as an auxiliary task.

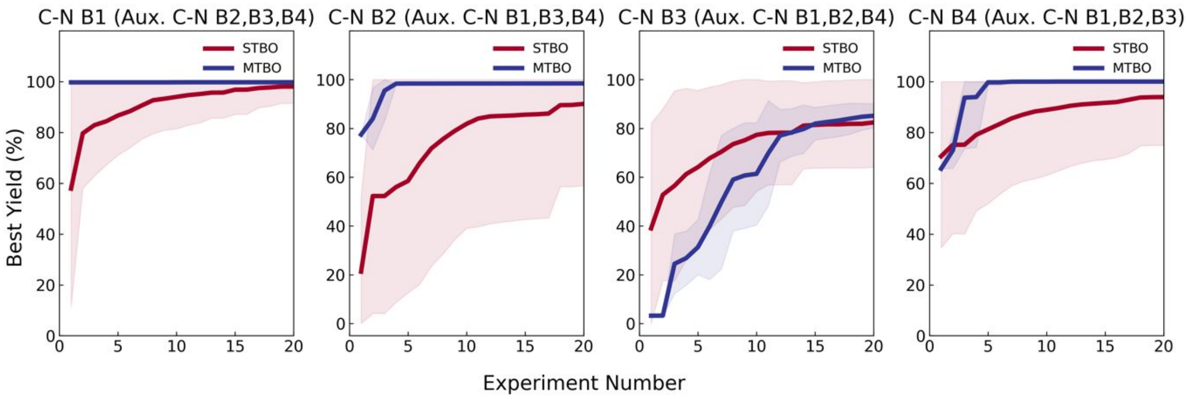

Figure S15: Comparison of the performance of single-task Bayesian optimization (STBO) and multi-task Bayesian optimization (MTBO) on C-N B1-B4 with all remaining C-N tasks for auxiliary training. The text above the plot represents the data used as an auxiliary task.

1.6 A note on literature data for optimization

During the preparation of this manuscript and the work discussed herein, the authors considered using data mined from reaction databases to improve performance of MTBO using multiple auxiliary tasks. However, when the authors attempted this, it did not generally work well for the following reasons:

- Typically, the variation in the literature is in the substrate, while in reaction optimization the variation is in agents (catalyst, reagent, solvent). Therefore, models trained on the literature tend to be unhelpful for reaction optimization. A recent paper from Wuistchik's team at Roche captures this issue well.[5]
- The broad applicability of using mined data for chemists is hindered by the lack of clean datasets from the literature. Obtaining a clean dataset from the literature takes a significant amount of work since synthetic chemistry data is not stored in a structured format. Even gathering data from reaxys (which is already heavily processed) requires several post-processing steps. For example, when the authors created a Suzuki coupling dataset from Reaxys, many of the reactions did not have yield (~50%) and 75% of the data with yield were duplicates.

2. C-H Activation Case Studies

2.1 General procedure for synthesis of product analytical standards

The general procedure as highlighted by Hennessy and Buchwald[6] was followed. An oven-dried 50 mL round-bottomed flask was equipped with a magnetic stir bar and air condenser. Palladium acetate (2 mol%), JohnPhos (4 mol%) and the chloroacetanilide substrate (5 mmol) was then charged, and the flask was evacuated and backfilled with nitrogen 3 times. Anhydrous triethylamine (1.05 mL, 10 mmol) was added, followed by anhydrous toluene (5 mL). The reaction mixture was heated at 80 °C and ran overnight, then diluted with ethyl acetate (50 mL). The mixture was filtered through a plug of celite, concentrated on a rotary evaporator, then purified by silica gel chromatography to give the oxindole product. For each case study, the starting material was purchased from J&H Chemical in 95 % purity. All other chemicals were purchased from Sigma Aldrich unless otherwise stated.

2.2 Experimental setup

The following experimental setup was used, as pictured in Figure S16, as described in the Methods section of the paper.

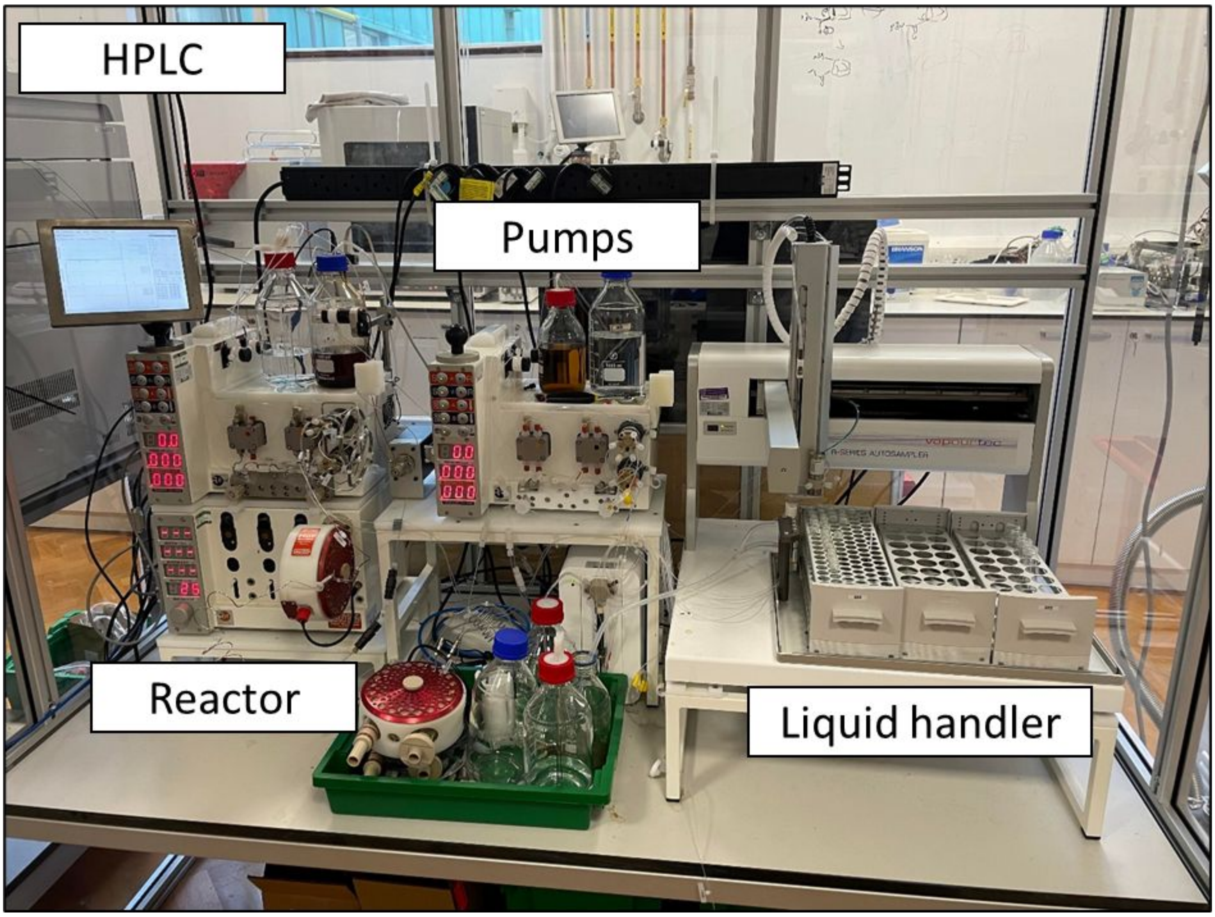

Figure S16: A photo of the experimental setup used for all C-H activation case studies.

Solubility studies were initially conducted to determine the maximum concentrations of each starting material and product in different solvents. This information would then be used to conclude the ideal

concentration of the starting material in each reaction (0.09 M) and the solvents of choice to help to prevent reactor clogging. In each study, a pre-weighed vial and a fixed amount of material was dosed with small amounts of solvent and stirred vigorously until the material was fully dissolved - the vial was then weighed, and the resulting concentration calculated. These maximum concentrations are reported in Table S1:

Table S1: The maximum concentration observed for each starting material and product from each case study in a number of solvents, as determined from solubility studies.

**Case study 1**								
	H_2O /M	Acetonitrile /M	THF /M	Methanol /M	Acetone /M	Chloroform /M	IPA /M	Toluene /M
**17**	0.06	5.44	4.65	4.22	4.88	5.47	2.06	4.15
**18**	0.03	0.76	0.85	0.30	0.73	2.43	0.31	0.46
**Case study 2**								
	H_2O /M	Acetonitrile /M	THF /M	Methanol /M	Acetone /M	Chloroform /M	IPA /M	Toluene /M
**19**	0.01	0.66	1.60	0.05	0.71	1.89	0.07	0.54
**20**	0.02	0.10	0.20	0.03	0.06	1.29	0.01	0.13
**Case study 3**								
	H_2O /M	Acetonitrile /M	THF /M	Methanol /M	Acetone /M	Chloroform /M	IPA /M	Toluene /M
**21**	0.02	2.62	2.29	0.28	2.51	2.24	0.24	0.70
**22**	0.01	0.08	0.08	0.03	0.06	0.19	0.01	0.06
**Case study 4**								
	H_2O /M	Acetonitrile /M	THF /M	Methanol /M	Acetone /M	Chloroform /M	IPA /M	Toluene /M
**23**	0.01	5.01	3.56	4.01	4.33	5.65	1.75	3.67
**24**	0.01	0.82	0.71	0.23	1.10	2.56	0.21	0.49

The weighing of the individual components into the sample vials differed for each of the case studies - these vials were used by the liquid handler as solution reservoirs. The vials 1 - 5 contained these differing components, whilst vials 6 - 25 remained constant throughout. 10 mL of reaction solution was present in each vial, which was each purged with nitrogen. The triethylamine and all solvents are anhydrous, and biphenyl was used as an internal standard. These concentrations were calculated so that a constant 0.09 M concentration of starting material in the reactor could be obtained through dilution, to enable a fair comparison between all conditions where mol% of catalyst was varied. The weights of all components are displayed in Table S2 and S3:

Table S2: Individual component weighing for each 'starting material' sample vial for the C-H activation case studies.

**Case study 1**				
Vial	*Solvent*	***17** mass /g*	*NEt_3 mass /g*	*Biphenyl mass /g*
1	Toluene	0.40	0.30	0.03
2	DMA	0.40	0.30	0.03
3	Acetonitrile	0.40	0.30	0.03
4	DMSO	0.40	0.30	0.03
5	NMP	0.40	0.30	0.03
**Case study 2**				
Vial	*Solvent*	***19** mass /g*	*NEt_3 mass /g*	*Biphenyl mass /g*
1	Toluene	0.89	0.30	0.03
2	DMA	0.89	0.30	0.03
3	Acetonitrile	0.89	0.30	0.03
4	DMSO	0.89	0.30	0.03
5	NMP	0.89	0.30	0.03
**Case study 3**				
Vial	*Solvent*	***21** mass /g*	*NEt_3 mass /g*	*Biphenyl mass /g*
1	Toluene	0.49	0.30	0.03

2	DMA	0.49	0.30	0.03
3	Acetonitrile	0.49	0.30	0.03
4	DMSO	0.49	0.30	0.03
5	NMP	0.49	0.30	0.03
**Case study 4**				
Vial	*Solvent*	***23** mass /g*	*NEt_3 mass /g*	*Biphenyl mass /g*
1	Toluene	0.45	0.30	0.03
2	DMA	0.45	0.30	0.03
3	Acetonitrile	0.45	0.30	0.03
4	DMSO	0.45	0.30	0.03
5	NMP	0.45	0.30	0.03

Table S3: Individual component weighing for each 'reagent' sample vial for the C-H activation case studies.

**All case studies**				
Vial	*Solvent*	*Ligand*	*$Pd(OAc)_2$ mass /g*	*Ligand mass /g*
6	Toluene	JohnPhos	0.045	0.119
7	DMA	JohnPhos	0.045	0.119
8	Acetonitrile	JohnPhos	0.045	0.119
9	DMSO	JohnPhos	0.045	0.119
10	NMP	JohnPhos	0.045	0.119
11	Toluene	SPhos	0.045	0.164
12	DMA	SPhos	0.045	0.164
13	Acetonitrile	SPhos	0.045	0.164
14	DMSO	SPhos	0.045	0.164
15	NMP	SPhos	0.045	0.164
16	Toluene	XPhos	0.045	0.194
17	DMA	XPhos	0.045	0.194
18	Acetonitrile	XPhos	0.045	0.194
19	DMSO	XPhos	0.045	0.194
20	NMP	XPhos	0.045	0.194
21	Toluene	DPEPhos	0.045	0.215
22	DMA	DPEPhos	0.045	0.215
23	Acetonitrile	DPEPhos	0.045	0.215
24	DMSO	DPEPhos	0.045	0.215
25	NMP	DPEPhos	0.045	0.215

2.3 Case study 1

An analytical standard for **18** was obtained using the general procedure, where LCMS analysis gave product m/z 166.15 and the ^{1}H NMR showed conversion to product as shown in Figure S17.

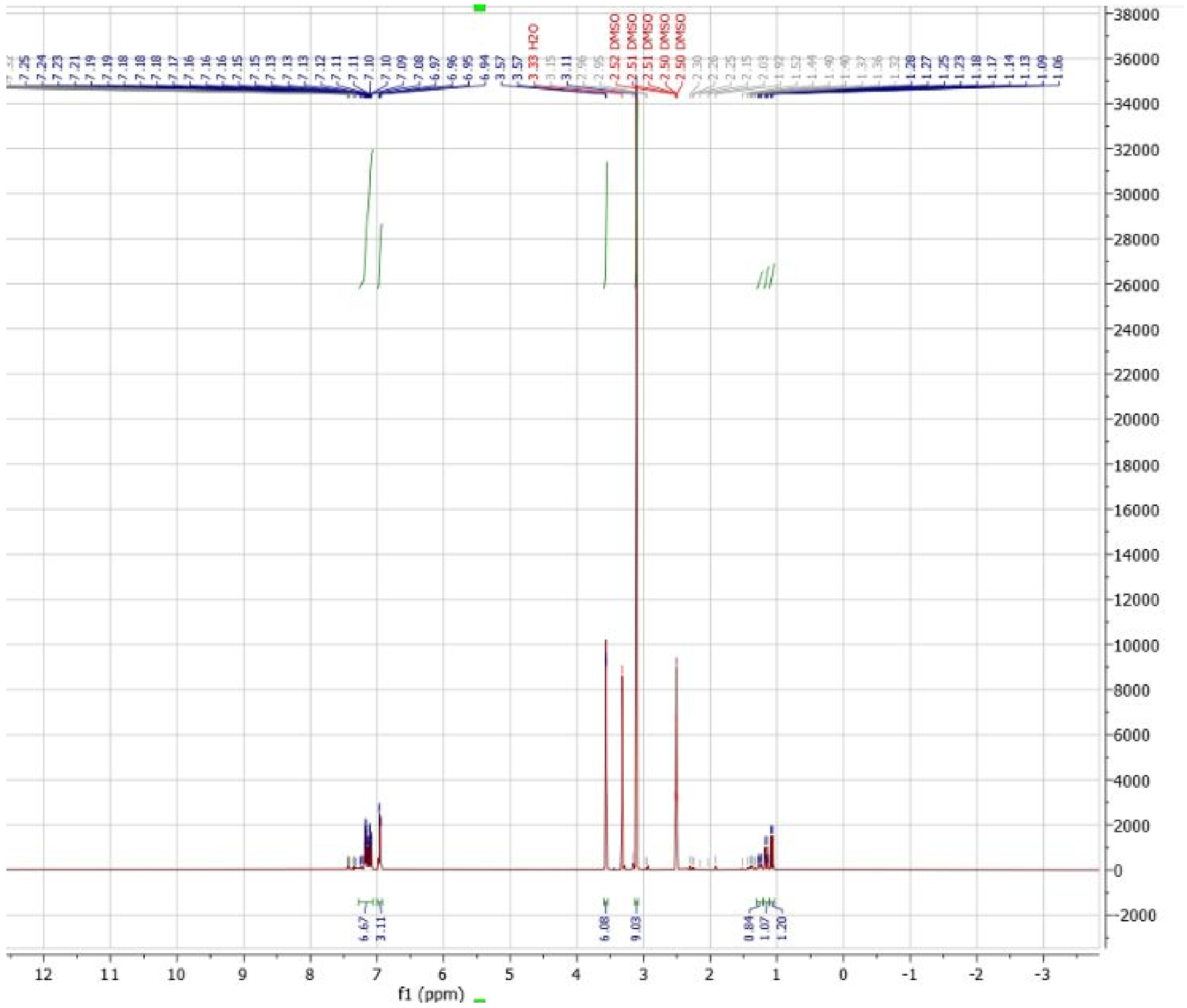

Figure S17: ^{1}H NMR for oxindole product **18**.

Table S4: All reaction data for the C-H activation case study 1.

	Conditions					
Type	Solvent	Ligand	Residence Time /min	Temp /°C	Mol%	Yield /%
Training	DMSO	DPEPhos	60	140	10	46.09
Training	DMSO	JohnPhos	45	70	3	1.31
Training	Toluene	DPEPhos	42	61	8	0.00
Training	DMA	JohnPhos	9	135	5	37.91
Training	MeCN	JohnPhos	11	134	2	16.87
Training	MeCN	JohnPhos	19	80	5	69.70
Training	NMP	JohnPhos	47	143	4	40.92
Training	DMSO	SPhos	29	122	4	0.00
Training	NMP	XPhos	52	112	8	55.69
Training	NMP	DPEPhos	26	53	6	0.00
Training	Toluene	XPhos	6	83	3	3.01
Training	MeCN	XPhos	39	90	9	6.33
Training	DMA	XPhos	51	104	9	16.11
Training	DMA	DPEPhos	22	67	4	4.04
Training	DMSO	XPhos	33	115	7	0.00
Training	Toluene	SPhos	19	57	8	0.00
Optimization	NMP	XPhos	60	102	10	56.23

Optimization	MeCN	JohnPhos	21	66	6	5.89
Optimization	Toluene	JohnPhos	53	141	8	26.36
Optimization	NMP	XPhos	60	123	9	42.37
Optimization	NMP	XPhos	53	89	9	74.64
Optimization	NMP	XPhos	46	88	9	46.62
Optimization	NMP	XPhos	60	85	8	72.40

2.4 Case study 2

An analytical standard for **20** was obtained using the general procedure, where LCMS analysis gave product m/z 409.30 and the ^{1}H NMR showed conversion to product in Figure S18. A pure product could not be obtained for this analytical standard and HPLC calibration was performed after quantitative NMR assays to determine %purity of product.

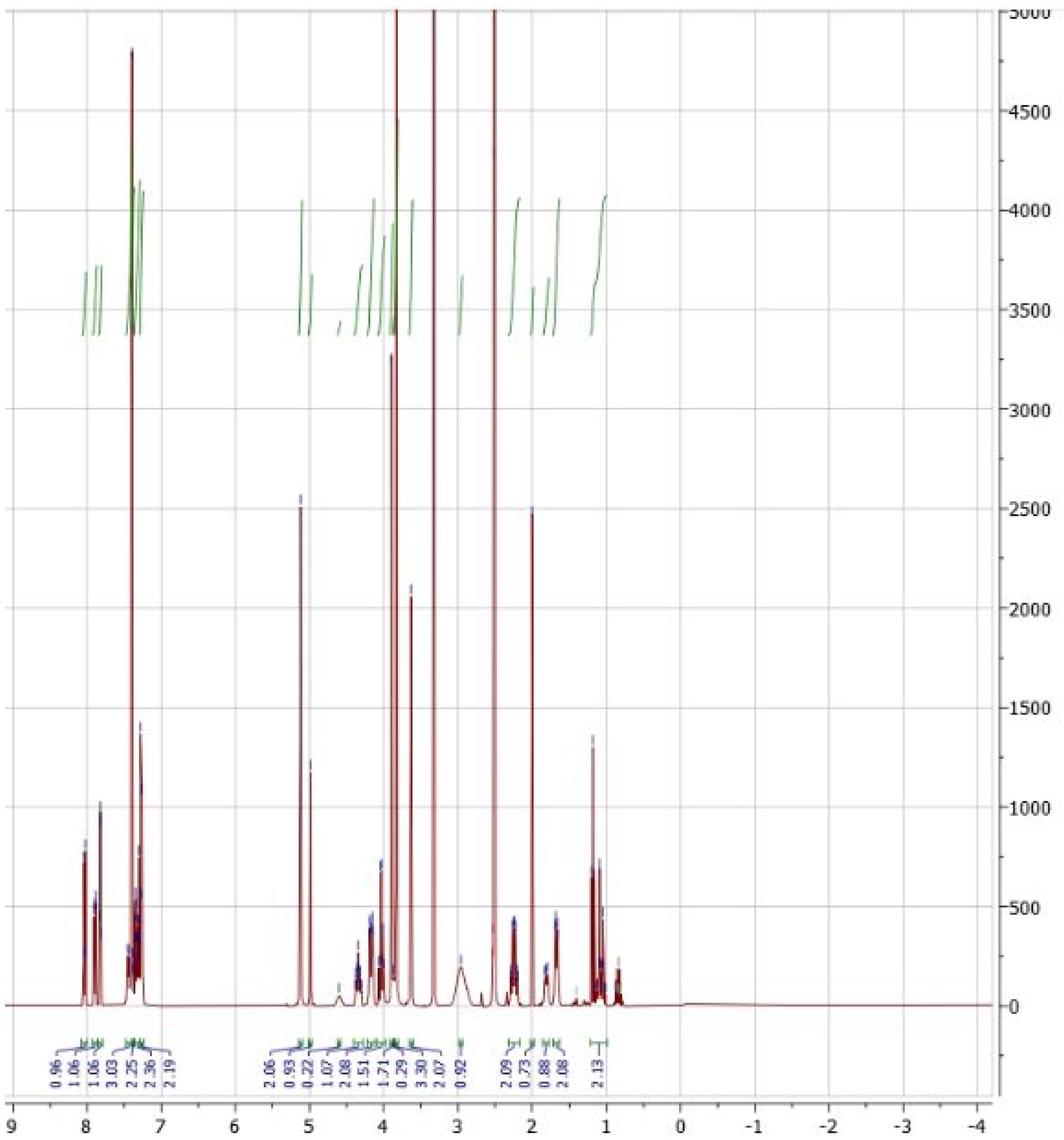

Figure S18: ^{1}H NMR for oxindole product **20**.

Table S5: All reaction data for the C-H activation case study 2.

	Conditions					
Type	Solvent	Ligand	Residence Time /min	Temp /°C	Mol%	Yield /%
Optimization	NMP	XPhos	19	75	4	14.78
Optimization	Toluene	DPEPhos	50	121	8	25.48
Optimization	MeCN	JohnPhos	21	59	7	11.10
Optimization	NMP	XPhos	58	91	9	30.98
Optimization	MeCN	JohnPhos	18	88	4	73.24
Optimization	MeCN	JohnPhos	27	93	4	76.92
Optimization	MeCN	JohnPhos	20	105	4	72.38
Optimization	MeCN	JohnPhos	40	100	8	80.11
Optimization	MeCN	JohnPhos	15	141	6	79.49
Optimization	MeCN	JohnPhos	19	117	5	84.70
Optimization	MeCN	JohnPhos	28	127	5	84.90

2.5 Case study 3

An analytical standard for **22** was obtained using the general procedure, where LCMS analysis gave product m/z 211.05 and the ^{1}H NMR showed conversion to product in Figure S19.

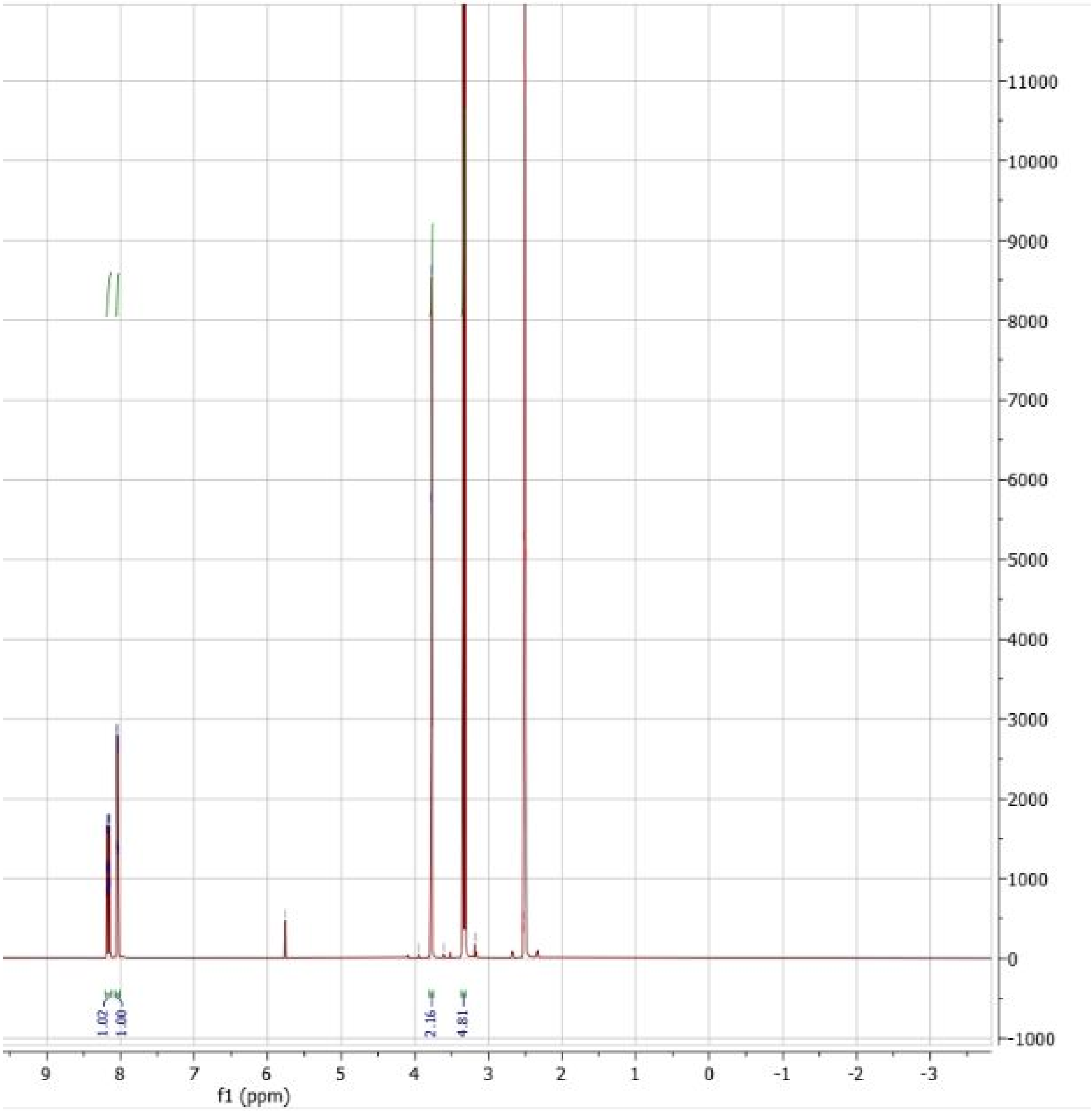

Figure S19: ^{1}H NMR for oxindole product **22**.

Table S6: All reaction data for the C-H activation case study 3.

	Conditions					
Type	Solvent	Ligand	Residence Time /min	Temp /°C	Mol%	Yield /%
Optimization	MeCN	JohnPhos	18	139	5	71.07
Optimization	NMP	XPhos	60	72	9	83.49
Optimization	NMP	XPhos	60	72	7	77.10
Optimization	NMP	XPhos	60	50	9	45.90
Optimization	NMP	XPhos	60	96	9	98.16

2.6 Case study 4

An analytical standard for **24** was purchased from Sigma Aldrich.

Table S7: All reaction data for the C-H activation case study 4.

	Conditions					
Type	Solvent	Ligand	Residence Time /min	Temp /°C	Mol%	Yield /%
Optimization	NMP	XPhos	60	96	9	6.29
Optimization	MeCN	JohnPhos	18	139	5	14.91
Optimization	Toluene	XPhos	28	125	3	1.63
Optimization	Toluene	SPhos	34	144	9	8.50
Optimization	Toluene	JohnPhos	55	140	6	9.95
Optimization	MeCN	JohnPhos	44	147	10	49.90
Optimization	DMSO	DPEPhos	59	143	9	81.97
Optimization	DMSO	DPEPhos	57	148	7	56.49
Optimization	DMSO	DPEPhos	60	150	10	82.21

2.7 Case study 5

An analytical standard for 1,7-dimethylindolin-2-one was obtained using the general procedure, where LCMS analysis gave product m/z 162.31 and the ^{1}H NMR showed conversion to product in Figure S20. A pure product could not be obtained for this analytical standard and HPLC calibration was performed after quantitative NMR assays to determine %purity of product.

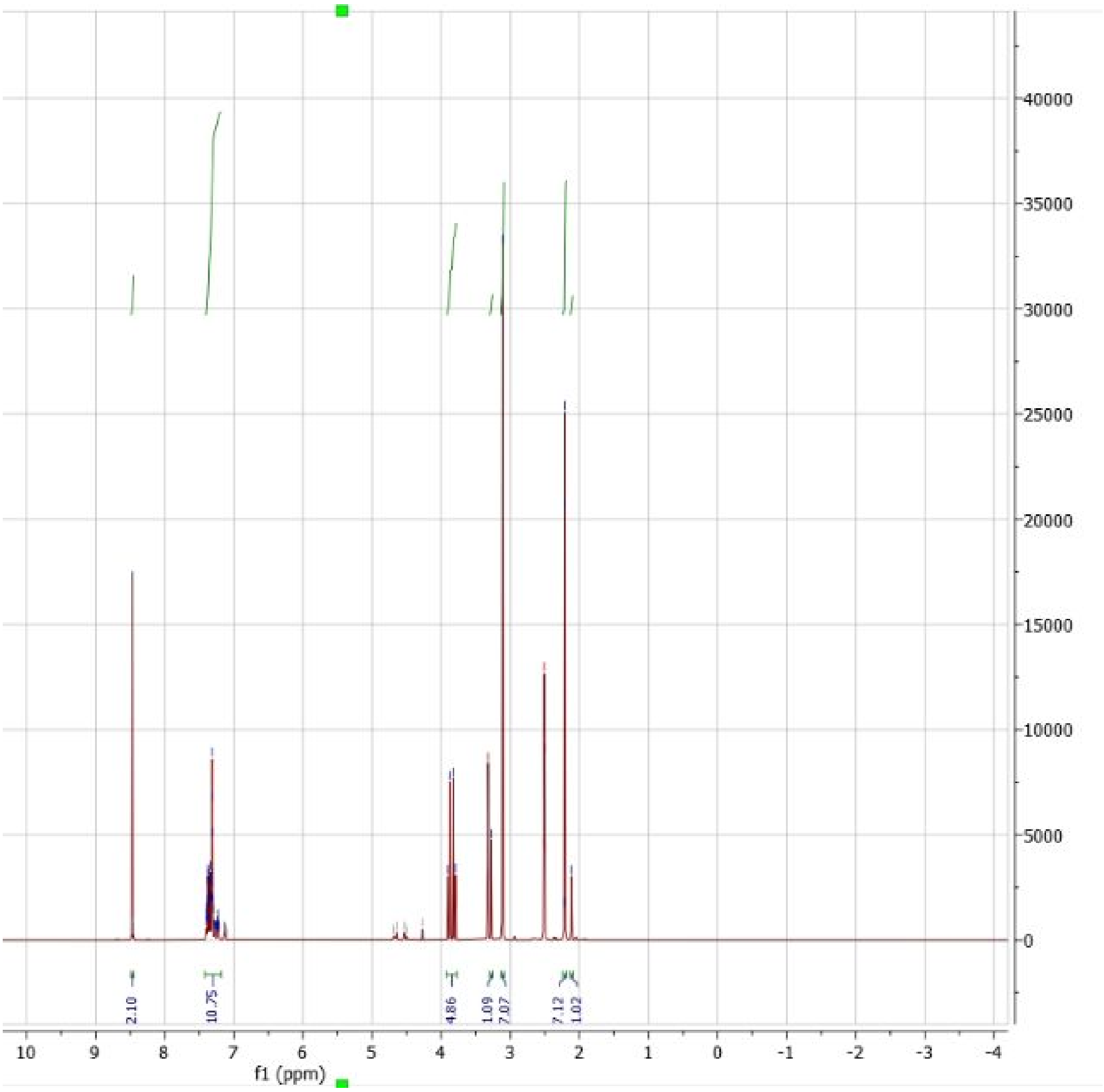

Figure S20: ^{1}H NMR for oxindole product 1,7-dimethylindolin-2-one.

Table S8: All reaction data for the C-H activation case study 5.

	Conditions					
Type	Solvent	Ligand	Residence Time /min	Temp /°C	Mol%	Yield /%
Optimization	MeCN	JohnPhos	18	139	5	17.23
Optimization	NMP	XPhos	60	72	9	3.85
Optimization	MeCN	JohnPhos	42	50	7	0.20
Optimization	NMP	DPEPhos	5	50	3	0.00
Optimization	Toluene	DPEPhos	54	150	4	1.56
Optimization	MeCN	JohnPhos	23	150	6	21.70
Optimization	MeCN	JohnPhos	25	132	6	21.50
Optimization	NMP	SPhos	36	141	9	13.70
Optimization	MeCN	JohnPhos	30	149	6	27.00
Optimization	MeCN	JohnPhos	37	150	6	29.30

3. Safety statement

During experimentation, there were no unexpected or unusually high safety hazards that were encountered.

4. References

(1) Kearnes, S. M.; Maser, M. R.; Wleklinski, M.; Kast, A.; Doyle, A. G.; Dreher, S. D.; Hawkins, J. M.; Jensen, K. F.; Coley, C. W. The Open Reaction Database. *J. Am. Chem. Soc.* 2021, **143**, 18820-18826.

(2) Baumgartner, L. M.; Dennis, J. M.; White, N. A.; Buchwald, S. L.; Jensen, K. F. Use of a Droplet Platform to Optimize Pd-Catalyzed C–N Coupling Reactions Promoted by Organic Bases. *Org. Process Res. Dev.* 2019, **23**, 1594-1601.

(3) Reizman, B. J.; Wang, Y.-M.; Buchwald, S. L.; Jensen, K. F. Suzuki–Miyaura Cross-Coupling Optimization Enabled by Automated Feedback. *React. Chem. Eng.* 2016, **1**, 658-666.

(4) Felton, K. C.; Rittig, J. G.; Lapkin, A. A. Summit: Benchmarking Machine Learning Methods for Reaction Optimisation. *Chem.: Methods.* 2021, **1**, 116-122.

(5) Fitzner, M.; Wuitschik, G.; Koller, R.; Adam, J.-M.; Schindler, T. Machine Learning C–N Couplings: Obstacles for a General-Purpose Reaction Yield Prediction. *ACS Omega.* 2023.

(6) Hennessy, E. J.; Buchwald, S. L. Synthesis of Substituted Oxindoles from A-Chloroacetanilides Via Palladium-Catalyzed C− H Functionalization. *J. Am. Chem. Soc.* 2003, **125**, 12084-12085.
